# Neuronal fatty acid-binding protein enhances autophagy and suppresses amyloid-β pathology in a *Drosophila* model of Alzheimer’s disease

**DOI:** 10.1371/journal.pgen.1011475

**Published:** 2024-11-19

**Authors:** Seokhui Jang, Byoungyun Choi, Chaejin Lim, Minkyoung Kim, Ji-Eun Lee, Hyungi Lee, Eunji Baek, Kyoung Sang Cho

**Affiliations:** 1 Department of Biological Sciences, Konkuk University, Seoul, Republic of Korea; 2 Korea Hemp Institute, Konkuk University, Seoul, Republic of Korea; National University of Singapore, SINGAPORE

## Abstract

Fatty acid-binding proteins (FABPs) are small cytoplasmic proteins involved in intracellular lipid transport and bind free fatty acids, cholesterol, and retinoids. FABP3, the major neuronal FABP in the adult brain, is upregulated in the CSF of patients with Alzheimer’s disease (AD). However, the precise role of neuronal FABPs in AD pathogenesis remains unclear. This study investigates the contribution of fabp, the *Drosophila* homolog of FABP3 and FABP7, to amyloid β (Aβ) pathology using a *Drosophila* model. Neuronal knockdown of *fabp* shortened the lifespan of flies and increased age-related protein aggregates in the brain. In an AD model, *fabp* knockdown in neurons increased Aβ accumulation and Aβ-induced neurodegeneration, whereas *fabp* overexpression ameliorated Aβ pathology. Notably, *fabp* overexpression stimulated autophagy, which was inhibited by the knockdown of *Eip75B*, the *Drosophila* homolog of the peroxisome proliferator-activated receptor (PPAR). The PPAR activator rosiglitazone restored autophagy impaired by *fabp* knockdown and reduced *fabp* knockdown-induced increased Aβ aggregation and cell death. Furthermore, knockdown of either *fabp* or *Eip75B* in the wing imaginal disc or adult fly brain reduced the expression of *Atg6* and *Atg8a*. Additionally, treatment of the *fabp* knockdown AD model flies with polyunsaturated fatty acids, such as docosahexaenoic acid or linoleic acid, partially alleviated cell death in the developing eye, restored impaired autophagy flux, reduced Aβ aggregation, and attenuated Aβ-induced cell death. Our results suggest that *Drosophila* fabp plays an important role in maintaining protein homeostasis during aging and protects neurons from Aβ-induced cell death by enhancing autophagy through the PPAR pathway. These findings highlight the potential importance of neuronal FABP function in AD pathogenesis.

## Author summary

Alzheimer’s disease (AD) is a neurodegenerative disease characterized by a gradual progression to severe cognitive impairment and dementia. Despite its high heritability, only a limited number of genes associated with AD have been identified. Understanding the role of disease-associated genes is critical to unraveling the pathology of AD. Given the abnormal protein aggregation seen in AD, genes that affect the degradation of aggregated proteins are expected to be important players. In this study, we focused on FABP, a lipid transport protein that has been observed at elevated levels in the cerebrospinal fluid of patients with AD. The specific role of this protein in neurons affected by AD remained elusive. Using a *Drosophila* model of AD, we found that reduced expression of *fabp* in neurons increased accumulation of amyloid β (Aβ) and exacerbated Aβ-induced neurodegeneration. Conversely, overexpression of *fabp* attenuated these phenotypes. Our results confirm that the protective effect of fabp is mediated through autophagy. In particular, we identified the involvement of the nuclear receptor PPAR in this process. We also demonstrated that neuronal fabp plays a critical role in protein aggregation and lifespan during aging. In conclusion, our study highlights the critical role of neuronal FABP as a regulator in AD pathology and aging. These findings contribute to a better understanding of the molecular mechanisms underlying AD and open avenues for potential therapeutic interventions.

## Introduction

Fatty acid-binding proteins (FABPs) are cytoplasmic proteins that act as molecular chaperones for lipids and play important roles in lipid transport and metabolism [1]. Various lipids, including polyunsaturated fatty acids (PUFAs), are ligands for FABPs. The apo form of FABP, which is not bound to a ligand, attaches to the lipid bilayer, while the ligand-bound form, holo-FABP, dissociates from the lipid bilayer and is transported by proteins such as importin α to deliver the ligand to the appropriate site [2]. There are at least nine classes of mammalian FABPs, expressed in various tissues and designated FABP1-9, according to the numerical nomenclature, or liver-FABP (L-FABP, FABP1), intestine-FABP (I-FABP, FABP2), and heart-FABP (H-FABP, FABP3), among others, according to the tissue in which their expression was first known [3,4]. In general, the expression of each FABP is not restricted to a limited number of tissues, and multiple types of FABPs are expressed simultaneously in a single tissue [1].

Three FABPs are expressed in the mammalian central nervous system (CNS): FABP3 (H-FABP), FABP5 (E-FABP), and FABP7 (B-FABP) [5,6]. As transporters of various lipids, FABPs serve various functions in neurons. They contribute to the supply and utilization of lipids in the brain, including docosahexaenoic acid (DHA), arachidonic acid, long-chain fatty acids, and retinoic acid, and are involved in functions such as membrane biogenesis and signaling [6]. The brain-expressed FABPs also play a role as intracellular carriers of endocannabinoids and epoxyeicosatrienoic acids to control their synaptic functions [7,8].

Previous studies emphasize the link between Alzheimer’s disease (AD) and FABPs. FABP3 levels are increased in the cerebrospinal fluid (CSF) of patients with AD compared to those in normal individuals [9–16], and it has been identified as an AD biomarker using multiplex platforms and validated in the Alzheimer’s Disease Neuroimaging Initiative database [17–20]. In addition, a study evaluating the proteome of the dorsolateral prefrontal cortex of the brain reported increased FABP7 levels in the brains of patients with AD with dementia than in those without cognitive symptoms [21]. Furthermore, the AD-associated variant apoE4 has been shown to cause FABP7 degradation and impairment of peroxisome proliferator-activated receptor γ (PPARγ)-dependent gene expression induced by FABP7 [22]. In a *Drosophila* AD model, neuronally expressed human *Aβ42* reduced fabp expression in the fly head, and either *Drosophila* fabp or mammalian Fabp7 rescued *Aβ42*-induced sleep fragmentation [23]. However, little is known about the function of FABP in the development and progression of AD.

In various tissues, FABPs transport lipid ligands to activate their nuclear receptor, the PPAR [6,24–27], which can act as sensors of lipid signaling in the context of autophagy regulation [28]. In bone marrow-derived macrophages, PPARs activate the transcription factor EB, which activates autophagy-related genes, such as *LAMP3* and *RAB7* [29]. PPARγ activation promotes PTEN transcription in various cells [30–32]. PTEN activation inhibits the PDK1-Akt pathway, which can in turn activate autophagy by inhibiting the activity of mTOR, a known autophagy inhibitor [28]. However, conflicting studies have reported that autophagy is activated in PPARγ knockout prostate epithelial cells [33] and that antagonizing PPARs in microglia promotes autophagy through the LKB1-AMPK signaling pathway [34], suggesting that autophagy regulation by PPARs may be tissue- or context-dependent. Similarly, the role of FABPs in autophagy remains unclear: in some studies, FABPs positively regulate autophagy [35–37], while in others, they act as negative regulators [38,39].

*FABP* genes are largely evolutionarily conserved among invertebrates [40]. *Drosophila* harbors one *fabp* gene in its genome, encoding a protein with the highest similarity to mouse Fabp7 (54% identical, 68% positive) [41]. According to the Aging Fly Cell Atlas, *Drosophila fabp* is expressed in various neurons and glial cells and its expression decreases with age in most cells [42]. In addition, although the whole-body overexpression of *fabp* increases the lifespan and resistance to oxidative stress in flies [43,44], but the underlying molecular mechanisms have not been studied. We previously reported that *Drosophila* fabp performs important functions in glial and neuronal development and affects behavioral phenotypes such as circadian rhythm and locomotor activity [45]. Furthermore, like its mammalian homolog, *Drosophila* fabp is involved in sleep [41], and the neuronal overexpression of *Drosophila fabp* or mammalian *Fabp7* alleviates *Aβ42*-induced sleep disruption by significantly increasing nighttime sleep and long consolidated sleep bouts [23].

FABP has been implicated not only in neurodegenerative diseases such as AD and PD but also in the aging process. However, the functional role of FABP in neuronal cells remains elusive. In this study, we investigated the impact of variations in *fabp* expression levels in *Drosophila* neuronal cells on brain aging and the responses to stressors such as Aβ accumulation. We found that fabp activates autophagy in a PPAR-dependent manner, mitigates age-related proteostasis breakdown, and suppresses Aβ accumulation, protecting neurons from cell death and neurodegeneration. Our findings underscore the critical role of FABP in maintaining brain health during aging and preventing Alzheimer’s pathology.

## Results

### Involvement of *Drosophila* neuronal fabp in aging and stress resistance

**Fig 1:**
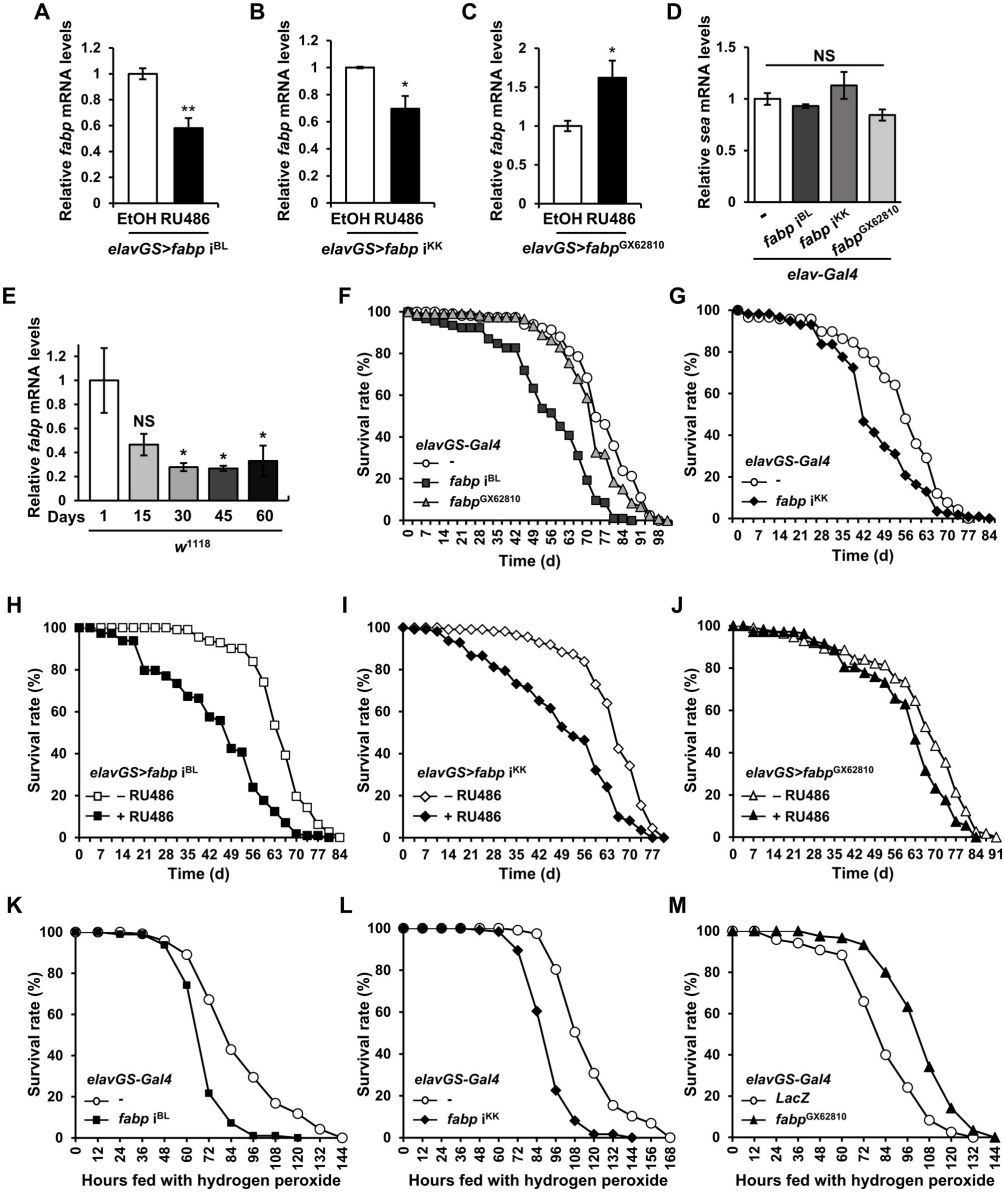
The role of neuronal fabp in systemic aging and susceptibility to ROS. (A-C) *fabp* mRNA levels in the heads of neuron-specific *fabp* knockdown (A, *elavGS*>*fabp* i^BL^; B, *elavGS*>*fabp* i^KK^) and neuron-specific *fabp* overexpression (C, *elavGS*>*fabp*^GX62810^) flies with or without 20 μM RU486 treatment for their entire lives (Student’s *t*-test, *p < 0.05, **p < 0.01; A and B, *N* = 3; C, *N* = 5). (D) *sea* mRNA levels in the heads of control (*elav-Gal4*/+), neuron-specific *fabp* knockdown (*elav>fabp* i^BL^ and *elav*>*fabp* i^KK^), and neuron-specific *fabp* overexpression (*elav*>*fabp*^GX62810^) flies (one-way ANOVA test, *N* = 3, NS, not significant). (E) *fabp* mRNA levels in the heads of *w*^1118^ during aging (Kruskal-Wallis test, *N* ≥ 3, *p < 0.05, NS, not significant). (F, G) Lifespan of control (*elavGS-Gal4*/+), neuron-specific *fabp* knockdown (*elavGS*>*fabp* i^BL^ and *elavGS*>*fabp* i^KK^), and neuron-specific *fabp* overexpression (*elavGS*>*fabp*^GX62810^) flies [Kaplan–Meier estimator and log-rank test; F, *n* ≥ 93, *p* = 0 (*elavGS-Gal4*/+ vs. *elavGS*>*fabp* i^BL^), *p* = 0.0214 (*elavGS-Gal4*/+ vs. *elavGS*>*fabp*^GX62810^); G, *n* ≥ 117, *p* = 0.0021 (*elavGS-Gal4*/+ vs. *elavGS*>*fabp* i^KK^)]. (H-J) Lifespan of *elavGS*>*fabp* i^BL^ (H), *elavGS*>*fabp* i^KK^ (I), and *elavGS*>*fabp*^GX62810^ flies (J) with or without 20 μM RU486 treatment for their entire lives [Kaplan–Meier estimator and log-rank test; H, *n* ≥ 112, *p* = 0 (−RU486 vs. +RU486); I, *n* ≥ 111, *p* = 0 (−RU486 vs. +RU486); J, *n* ≥ 108, *p* = 0.0009 (−RU486 vs. +RU486)]. (K-M) Survival rate of control (*elavGS-Gal4*/+ or *elavGS*>*LacZ*), neuron-specific *fabp* knockdown (*elavGS*>*fabp* i^BL^ and *elavGS*>*fabp* i^KK^), and neuron-specific *fabp* overexpression (*elavGS*>*fabp*^GX62810^) flies under oxidative stress conditions [Kaplan–Meier estimator and log-rank test; K, *n* ≥ 97, *p* = 0 (*elavGS-Gal4*/+ vs. *elavGS*>*fabp* i^BL^); L, *n* ≥ 117, *p* = 0 (*elavGS-Gal4*/+ vs. *elavGS*>*fabp* i^KK^); M, *n* = 120, *p* = 0 (*elavGS>LacZ* vs. *elavGS*>*fabp*^GX62810^)]. All flies in (F, G, K, L, M) were grown in medium containing 20 μM RU486 for their entire lives. All data are expressed as mean ± SEM.

Given that *Drosophila* fabp is associated with aging and AD pathology [23,43,44], we investigated its role in fly neurons under conditions of aging and oxidative stress, using two *fabp* RNAi lines and one overexpression line. As we have shown in a previous study [45], *fabp* is critical for neurodevelopment, and flies expressing *fabp* RNAi using *elav-Gal4* showed lethality at the larval stage. Therefore, we used *elavGS-Gal4*, an inducible ’GeneSwitch’ Gal4 system, to regulate the *fabp* gene expression in neurons under conditions that do not result in developmental abnormalities and observed the consequences of this regulation. Expectedly, *fabp* RNAi reduced *fabp* expression, while *fabp* overexpression significantly increased *fabp* levels, as demonstrated by quantitative RT-PCR (Fig 1A–1C). Conversely, these manipulations did not affect the expression of *scheggia* (*sea*), a neighboring gene with partially overlapping alternative splicing forms of *fabp* (Fig 1D). According to the recently published Aging Fly Cell Atlas [42], *Drosophila fabp* expression decreases with age in most cell types, so we examined *fabp* expression level with aging and found a consistent decrease in *fabp* expression with aging in the fly head (Fig 1E).

We then examined the impact of changes in *fabp* expression on *Drosophila* lifespan and susceptibility to oxidative stress. Intriguingly, in contrast to previous findings that global *fabp* overexpression prolonged lifespan [43,44], both the neuron-specific downregulation and overexpression of *fabp* shortened the lifespan in our study (Fig 1F and 1G and S1 and S2 Tables). These results were consistent even under controlled gene expression with or without RU486 administration (+RU486 vs. −RU486; Fig 1H–1J and S3–S5 Tables), suggesting that the effect of the *fabp* gene on lifespan we observed is not driven by genetic background. However, different results emerged under oxidative stress conditions. Specifically, while the neuron-specific suppression of *fabp* decreased survival compared to that in controls in hydrogen-peroxide-induced oxidative stress situations, *fabp* overexpression increased the survival rate (Fig 1K–1M and S6–S8 Tables). These findings indicate that *fabp* may have a neuroprotective function in the context of oxidative stress, in contrast to its effects in the aging process.

### The critical role of fabp in proteostasis during aging

**Fig 2:**
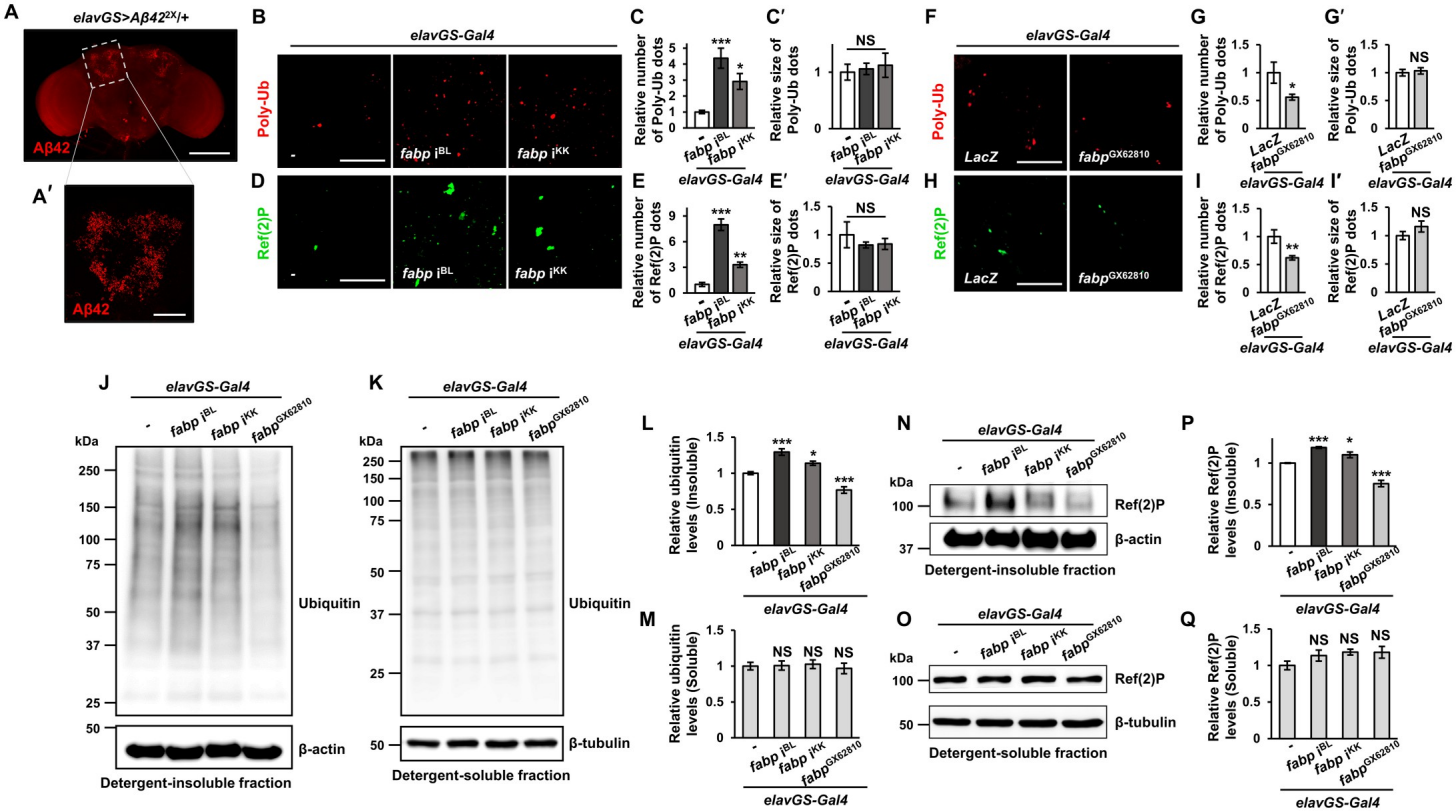
The role of neuronal fabp in proteostasis in the brain. (A) The brain of a *Aβ42*-expressing fly. (A′) The dotted rectangular region of (A), which is the area of the brain with Kenyon cell bodies where Aβ mainly accumulates. (B) Representative confocal images showing the polyubiquitination (Poly-Ub) in the brains of control (*elavGS-Gal4*/+) and neuron-specific *fabp* knockdown (*elavGS>fabp* i^BL^ and *elavGS>fabp* i^KK^) flies. The observations for the region shown in (A′). (C, C′) Quantification of the number (C) and size (C′) of Poly-Ub aggregates in the brains of indicated flies (C, one-way ANOVA test; C′, Kruskal-Wallis test; *n* = 7, *p < 0.05, ***p < 0.001, NS, not significant). (D) Representative confocal images showing the Ref(2)P aggregates in the brains of indicated flies. The observations for the region shown in (A′). (E, E′) Quantification of number (E) and size (E′) of Ref(2)P aggregates in the brains of indicated flies (one-way ANOVA test, *n* ≥ 7, **p < 0.01, ***p < 0.001, NS, not significant). (F) Representative confocal images showing the Poly-Ub aggregates in the brains of control (*elavGS*>*LacZ*) and neuron-specific *fabp* overexpression (*elavGS*>*fabp*^GX62810^) flies. (G, G′) Quantification of the number (G) and size (G′) of Poly-Ub aggregates in the brains of indicated flies (Student’s *t*-test, *n* = 14, *p < 0.05, NS, not significant). (H) Representative confocal images showing the Ref(2)P aggregates in the brains of indicated flies. (I, I′) Quantification of number (I) and size (I′) of Ref(2)P aggregates in the brains of indicated flies (Student’s *t*-test, *n* ≥ 10, **p < 0.01, NS, not significant). (J, K) Western blot analyses of ubiquitinated protein levels in detergent-insoluble (J) and detergent-soluble (K) fractions of the head extract from indicated flies. (L, M) Quantification of the ubiquitinated protein levels in detergent-insoluble (L) and detergent-soluble (M) fractions of head extract from indicated flies (one-way ANOVA test, *N* = 8, *p < 0.05, ***p < 0.001, NS, not significant). (N, O) Western blot analyses of Ref(2)P protein levels in detergent-insoluble (N) and detergent-soluble (O) fractions of the head extract from indicated flies. (P, Q) Quantification of the Ref(2)P protein levels in detergent-insoluble (P) and detergent-soluble (Q) fractions of head extract from indicated flies (one-way ANOVA test, *p < 0.05, ***p < 0.001, NS, not significant; P, *N* ≥ 4; Q, *N* = 4). All flies were grown in medium containing 20 μM RU486 for their entire lives. All data are expressed as mean ± SEM. Scale bars: 25 μm (B, D, F, H), 50 μm (A′), 200 μm (A).

We also examined the effect of the *fabp* gene on the age-related accumulation of polyubiquitinated protein aggregates, often used as indicators of aging in the *Drosophila* brain, and Ref(2)P puncta, a cargo receptor for autophagosomes, by performing immunohisto-chemistry with anti-polyubiquitin and anti-Ref(2)P antibodies, respectively. In particular, we focused on the Kenyon cell body and its surroundings, which are involved in fly memory and where Aβ accumulates (Fig 2A and 2A′). In the brains of 20-day-old *fabp*-knockdown flies, the levels of polyubiquitinated protein aggregates and Ref(2)P puncta increased than those in the brains of control flies, while a relative decrease was observed in the brains of *fabp*-overexpressing flies (Fig 2B–2I′). The quantitative changes of these proteins in *Drosophila* heads after the regulation of *fabp* expression were further validated by western blot analysis (Fig 2J–2Q). Consistent with the immunohistochemistry results, knockdown of *fabp* increased the amount of these proteins in the detergent-insoluble fraction, whereas overexpression of *fabp* decreased it. These results suggest an important role for fabp in maintaining proteostasis in the neuronal context.

### Neuroprotective role of neuronal fabp against Aβ pathology in *Drosophila*

**Fig 3:**
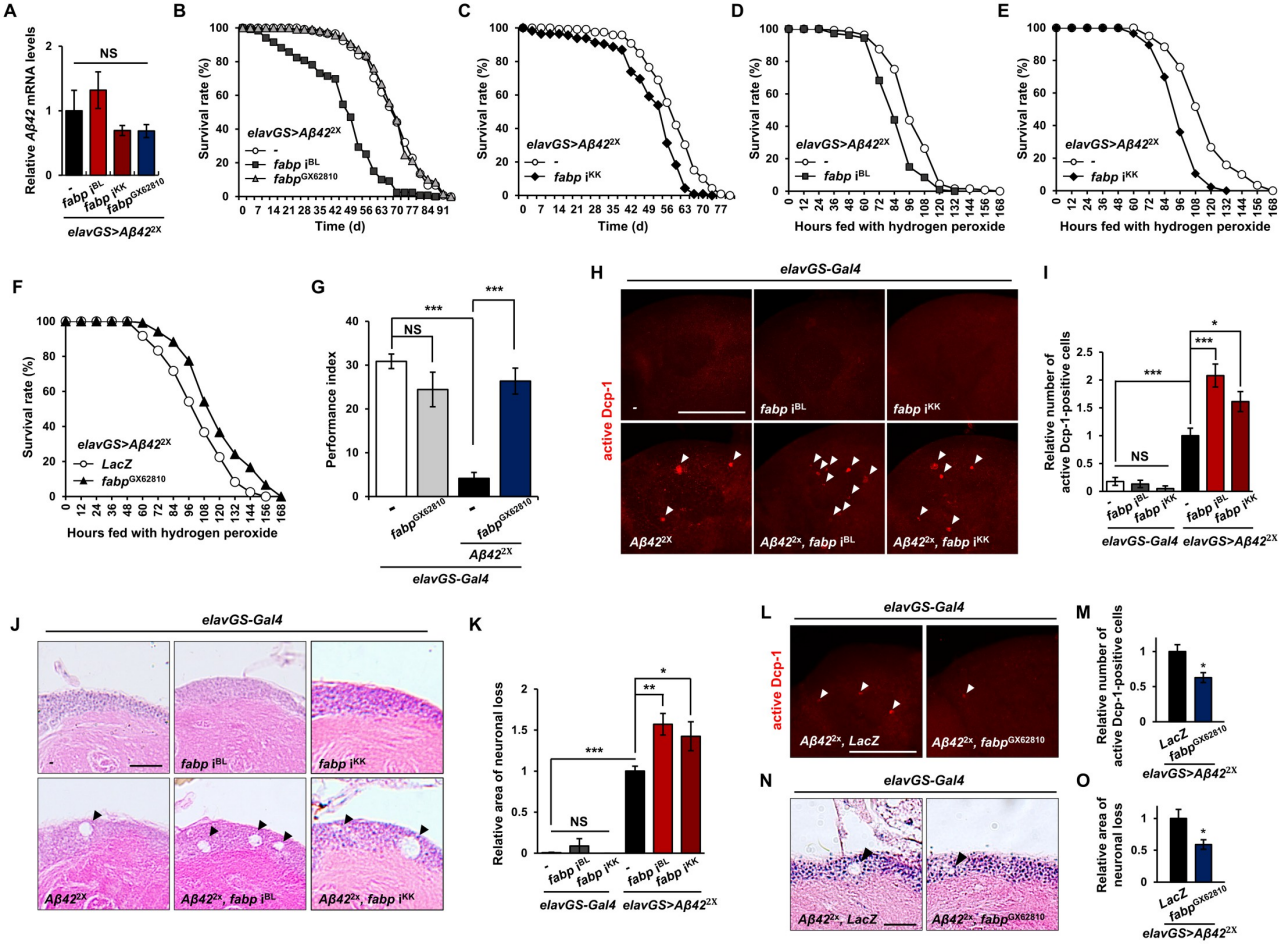
Protective role of fabp against Aβ pathology. (A) Comparison of *Aβ42* mRNA levels on heads of *Aβ42*-expressing flies with *fabp* knockdown (*elavGS*>*Aβ42*^2x^, *fabp* i^BL^ and *elavGS*>*Aβ42*^2x^, *fabp* i^KK^) or *fabp* overexpression (*elavGS*>*Aβ42*^2x^, *fabp*^GX62810^) in neurons with control flies (*elavGS*>*Aβ42*^2x^/+) (one-way ANOVA test, *N* ≥ 3, NS, not significant). (B, C) Lifespan of control flies (*elavGS*>*Aβ42*^2x^/+) and *Aβ42*-expressing flies with neuron-specific *fabp* knockdown (*elavGS*>*Aβ42*^2x^, *fabp* i^BL^ and *elavGS*>*Aβ42*^2x^, *fabp* i^KK^) or neuron-specific *fabp* overexpression (*elavGS*>*Aβ42*^2x^, *fabp*^GX62810^) [Kaplan–Meier estimator and log-rank test; B, *n* ≥ 117, *p* = 0 (*elavGS*>*Aβ42*^2x^/+ vs. *elavGS*>*Aβ42*^2x^, *fabp* i^BL^), *p* = 0.7601 (*elavGS*>*Aβ42*^2x^/+ vs. *elavGS*>*Aβ42*^2x^, *fabp*^GX62810^); C, *n* ≥ 115, *p* = 1.2e-7 (*elavGS*>*Aβ42*^2x^/+ vs. *elavGS*>*Aβ42*^2x^, *fabp* i^KK^)]. (D-F) Survival rate of control flies (*elavGS*>*Aβ42*^2x^/+ and *elavGS*>*Aβ42*^2x^, *LacZ*) and *Aβ42*-expressing flies with neuron-specific *fabp* knockdown (*elavGS*>*Aβ42*^2x^, *fabp* i^BL^ and *elavGS*>*Aβ42*^2x^, *fabp* i^KK^) or neuron-specific *fabp* overexpression (*elavGS*>*Aβ42*^2x^, *fabp*^GX62810^) under oxidative stress conditions [Kaplan–Meier estimator and log-rank test; D, *n* ≥ 107, *p* = 4.2e-8 (*elavGS*>*Aβ42*^2x^/+ vs. *elavGS*>*Aβ42*^2x^, *fabp* i^BL^); E, *n* ≥ 86, *p* = 0 (*elavGS*>*Aβ42*^2x^/+ vs. *elavGS*>*Aβ42*^2x^, *fabp* i^KK^); F, *n* = 120, *p* = 7.3e-6 (*elavGS*>*Aβ42*^2x^, *LacZ* vs. *elavGS*>*Aβ42*^2x^, *fabp*^GX62810^)]. (G) Aversive associative memory performance at 90 s after training of 20-day-old flies (one-way ANOVA test, *N* ≥ 5, ***p < 0.001, NS, not significant). (H-O) Effect of neuron-specific *fabp* knockdown (H-K) and overexpression (L-O) on Aβ-induced apoptosis (H, I, L, M) and neurodegeneration (J, K, N, O). (H, J, L, N) Representative images of confocal showing the active Dcp-1 immunostaining (H, L) and the H&E-stained frontal brain sections showing neurodegeneration (J, N) in the brains of indicated flies. (I, K, M, O) Quantification of the relative number of active Dcp-1-positive cells (I, M) and the area of vacuoles (K, O) in the brains of indicated flies (*p < 0.05, **p < 0.01, ***p < 0.001, NS, not significant; I and K, one-way ANOVA test; M and O, Student’s *t*-test; I, *n* ≥ 9; K, *n* = 10; M, *n* ≥ 8; O, *n* ≥ 15). All flies were grown in medium containing 20 μM RU486 for their entire lives. All data are expressed as mean ± SEM. Scale bars: 20 μm (J, N) and 100 μm (H, L). White arrow heads indicate active Dcp-1-positive cells, and black arrow heads indicate vacuoles.

While the overexpression of *Drosophila fabp* rescues fragmented sleep in a fly model of AD [23], its protective effects in general Aβ pathology remain unknown. To investigate the impact of altered *fabp* expression in fly neurons on Aβ pathology, we first assessed whether changes in *fabp* expression affect Aβ transgene expression. Our findings revealed that co-expression of *fabp* RNAi and *Aβ42* or *fabp* and *Aβ42* did not affect *Aβ42* gene expression (Fig 3A). However, *fabp* RNAi expression in the presence of Aβ (Fig 3B and 3C, S9 and S10 Tables) reduced *Drosophila* lifespan similarly to *fabp* RNAi expressed alone (Fig 1F–1I). These results were consistent even under controlled gene expression with or without RU486 administration (S1 Fig and S11–S13 Tables). Nevertheless, the extent of lifespan reduction resulting from the co-expression of *fabp* RNAi and *Aβ42* was comparable to the sum of the reductions observed in lifespan following the individual expressions, indicating that the functions of fabp and Aβ in regulating lifespan are additive rather than specifically interacting (Figs 1H and 1I and S1, S3, S4 and S11–S13 Tables).

Although neuron-specific *fabp* overexpression reduced the lifespan of flies in the absence of Aβ (Fig 1F and 1J, S1–S5 Tables), it did not affect the lifespan of AD model flies expressing Aβ in neurons (Fig 3B and S9 Table). This suggests a relative benefit of fabp in the presence of Aβ compared to that in the absence of Aβ.

Furthermore, when we examined the effect of neuronal *fabp* expression on the oxidative stress susceptibility of *Aβ42*-expressing flies, *fabp* RNAi decreased resistance to oxidative stress, whereas the overexpression increased it (Fig 3D–3F and S14–S16 Tables).

Next, we investigated the effect of neuronal *fabp* expression levels on short-term memory in a *Drosophila* AD model. As previously reported [46], flies expressing *Aβ42* showed a pronounced memory impairment compared to controls (Fig 3G). The overexpression of *fabp* alone in neurons did not affect short-term memory, but its co-expression with *Aβ42* reversed Aβ-induced memory impairment and restored the memory to near to the control levels (Figs 3G and S2).

Given that neuronal *Aβ42* expression induces histopathological changes in the *Drosophila* brain similar to those observed in the brains of patients with AD [47,48], we investigated the influence of neuronal *fabp* on Aβ-induced histological changes in the fly brain. First, to readily determine if *fabp* is likely to affect Aβ-induced cytotoxicity, we examined its effect on the *Aβ42*-induced rough-eye phenotype. When *Aβ42* is ectopically expressed in the fly eye, a modest rough eye phenotype and black spots are observed (S3A Fig upper left panel). The concurrent knockdown of *fabp* along with *Aβ42* expression resulted in the death of nearly half of the posterior segment photoreceptor cells, whereas overexpression of *fabp* almost completely abolished the black spots in *Aβ42*-expressing eye (S3A Fig upper panels). The protective effect of *fabp* was also confirmed at the cellular level in the developing eye by immunohistochemistry with an antibody against the activated form of *Drosophila* caspase, active Dcp-1 (S3A Fig, lower panels and S3B Fig). Then, we examined the effect of *fabp* expression levels on Aβ-induced brain cell apoptosis and neurodegeneration by immunohistochemistry and brain sections followed by H&E staining. Without Aβ, neither *fabp* knockdown nor overexpression induced cell death or neurodegeneration in the brain (Fig 3H–3K). However, when *Aβ42* was expressed in neurons, *fabp* knockdown or mutant significantly enhanced Aβ-induced apoptosis (Figs 3H, 3I and S4A–S4C), whereas *fabp* overexpression attenuated it (Fig 3L and 3M). Correspondingly, *fabp* knockdown significantly increased the area of vacuoles generated by Aβ-induced neurodegeneration (Fig 3J and 3K), whereas *fabp* overexpression reduced it (Fig 3N and 3O). These results suggest that neuronal fabp protects neurons from Aβ toxicity.

**Fig 4:**
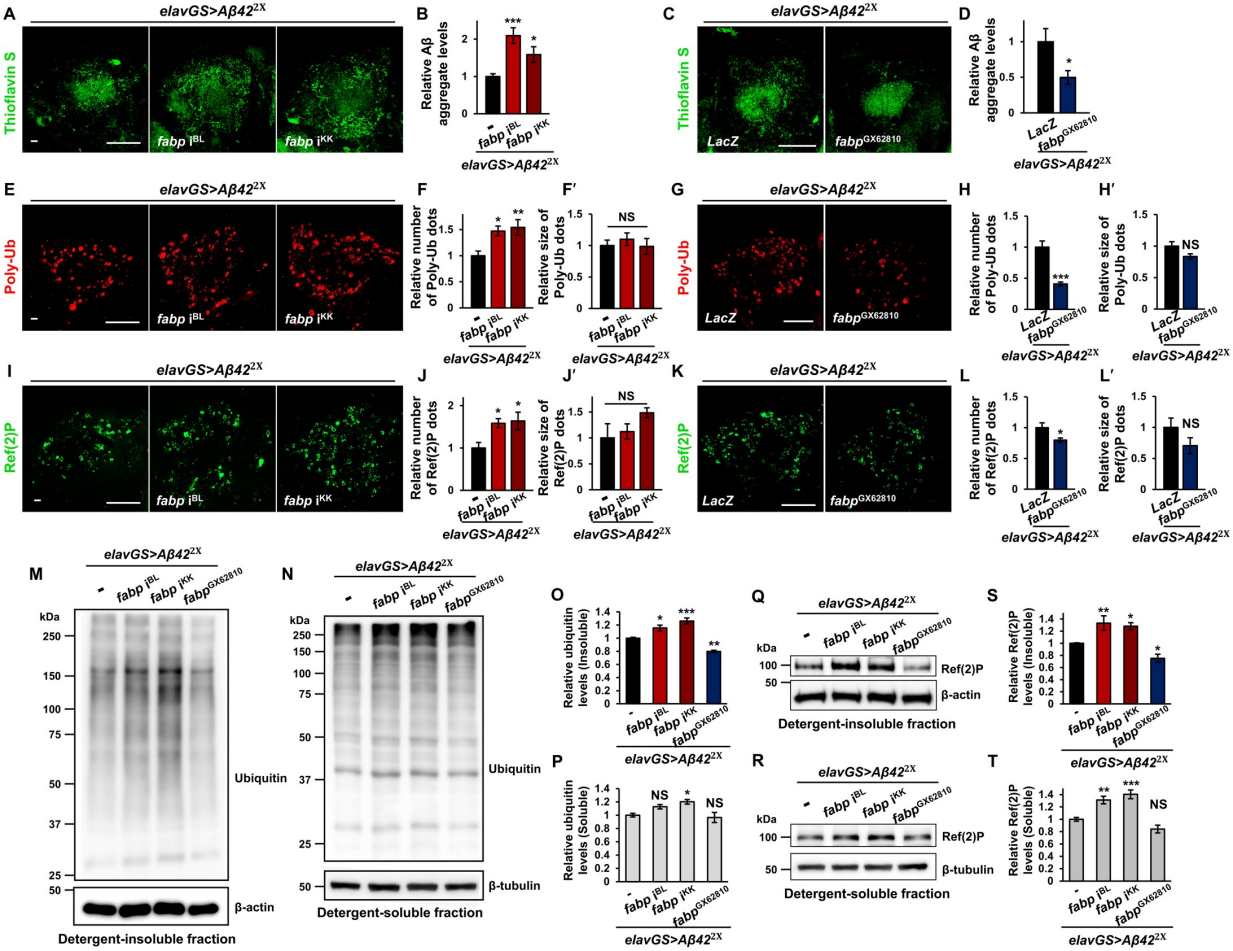
Role of fabp in Aβ aggregation and proteostasis in the brain of *Drosophila* AD model. (A-D) Effect of neuron-specific *fabp* knockdown (A, B) and overexpression (C, D) on Aβ aggregation. (A, C) Thioflavin S staining showing the Aβ aggregation in the brains of control (*elavGS*>*Aβ42*^2x^/+ and *elavGS*>*Aβ42*^2x^, *LacZ*), neuron-specific *fabp*-knockdown (*elavGS*>*Aβ42*^2x^, *fabp* i^BL^ and *elavGS*>*Aβ42*^2x^, *fabp* i^KK^), and neuron-specific *fabp*-overexpression (*elavGS*>*Aβ42*^2x^, *fabp*^GX62810^) flies. (B, D) Quantification of Aβ aggregate intensity in the brains of indicated flies (B, one-way ANOVA test, *n* = 10, *p < 0.05, ***p < 0.001; D, Student’s *t*-test, *n* ≥ 12, *p < 0.05). (E-H′) Effect of neuron-specific *fabp* knockdown (E-F′) and overexpression (G-H′) on the accumulation of polyubiquitin-positive protein aggregates (Poly-Ub). (E, G) Immunostaining of Poly-Ub in the brains of indicated flies. (F-F′, H-H′) Quantification of the number (F, H) and size (F′, H′) of Poly-Ub aggregates in the brains of indicated flies (*p < 0.05, **p < 0.01, ***p < 0.001, NS, not significant; F, one-way ANOVA test, *n* = 10; F′, Kruskal-Wallis test, *n* = 10; H and H′, Student’s *t*-test, *n* = 9). (I-L′) Effect of neuron-specific *fabp* knockdown (I-J′) and overexpression (K-L′) on the accumulation of Ref(2)P aggregates. (I, K) Immunostaining of Ref(2)P aggregates in the brains of indicated flies. (J-J′, L-L′) Quantification of the number (J, L) and size (J′, L′) of Ref(2)P aggregates in the brains of indicated flies (*p < 0.05, NS, not significant; J, one-way ANOVA test, *n* = 8; J′, Kruskal-Wallis test, *n* = 8; L-L′, Student’s *t*-test, *n* = 9). (M, N) Western blot analyses of ubiquitinated protein levels in detergent-insoluble (M) and detergent-soluble (N) fractions of the head extract from indicated flies. (O, P) Quantification of the relative ubiquitinated protein levels in detergent-insoluble (O) and detergent-soluble (P) fractions (one-way ANOVA test, *p < 0.05, **p < 0.01, ***p < 0.001, NS, not significant; O, *N* ≥ 4; P, *N* = 4). (Q, R) Western blot analyses of Ref(2)P protein levels in detergent-insoluble (Q) and detergent-soluble (R) fractions of the heads from indicated flies. (S, T) Quantification of the relative Ref(2)P protein levels in detergent-insoluble (S) and detergent-soluble (T) fractions (one-way ANOVA test, *p < 0.05, **p < 0.01, ***p < 0.001, NS, not significant; S, *N* ≥ 6; T, *N* = 6). All flies were grown in medium containing 20 μM RU486 for their entire lives and aged for 20 days. All data are expressed as mean ± SEM. Scale bars: 50 μm.

As fabp can inhibit protein aggregation (Fig 2), we next investigated whether the neuroprotective effect of fabp against Aβ was due to its ability to limit Aβ accumulation. To address this, we examined the effect of fabp on Aβ aggregation using thioflavin S staining. Our results showed that *fabp* knockdown or mutant significantly increased the accumulation of Aβ aggregates (Figs 4A, 4B, S4D and S4E), whereas *fabp* overexpression resulted in a significant reduction (Fig 4C and 4D). Similarly, in the *Drosophila* AD model brain, the ubiquitin-positive protein aggregates and Ref(2)P puncta were significantly increased following *fabp* knockdown but decreased following *fabp* overexpression (Fig 4E–4L′). Western blot analyses also supported the immunohistochemistry results (Fig 4M–4T). These results suggest that fabp attenuates Aβ pathology by reducing Aβ deposition.

### *Drosophila* fabp is a modulator of autophagy

**Fig 5:**
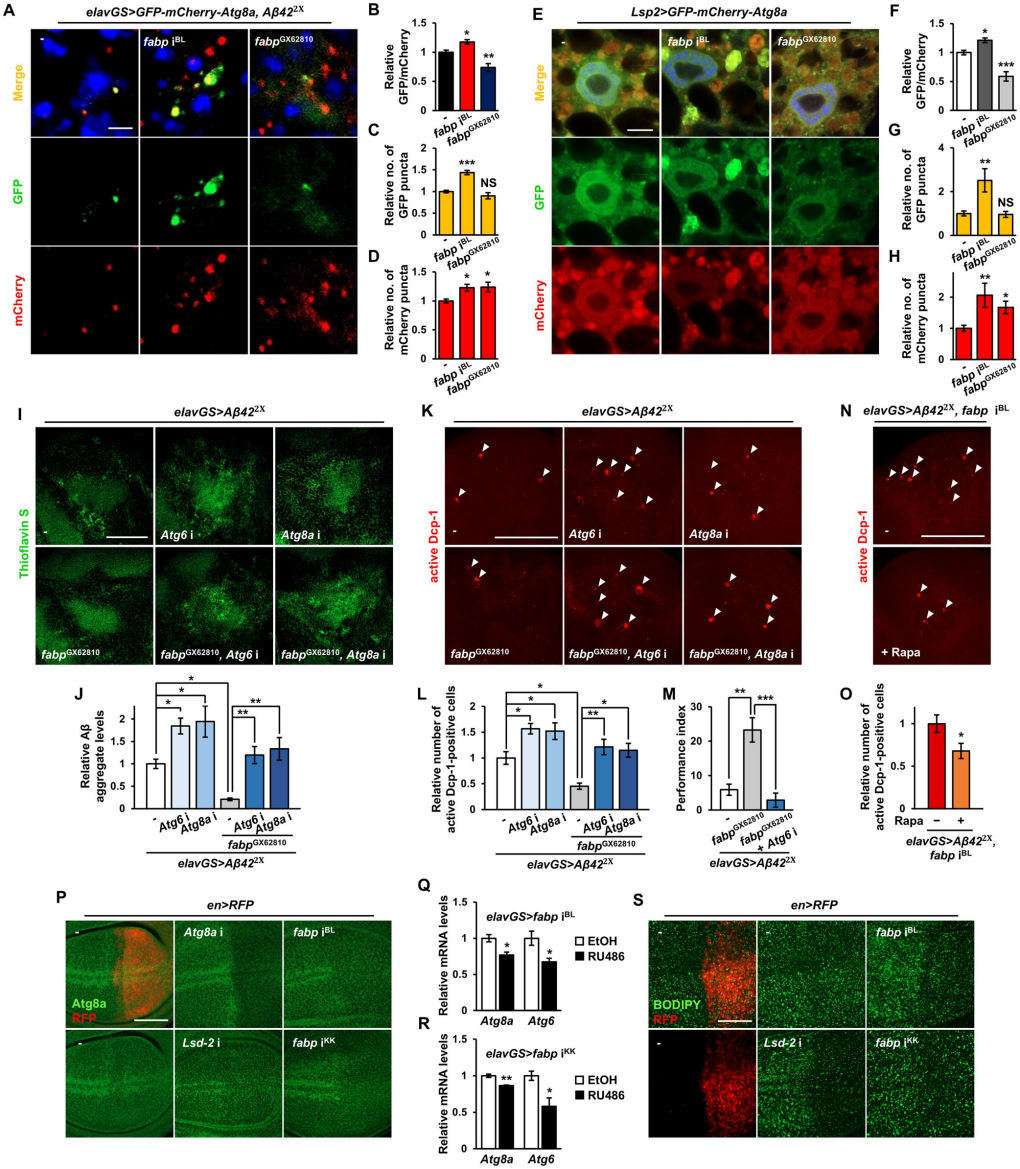
Fabp affects Aβ pathology via autophagy regulation. (A) Representative confocal images showing GFP-mCherry-Atg8a puncta in the brains of control flies (*elavGS*>*GFP-mCherry-Atg8a*, *Aβ42*^2x^/+) and *Aβ42*-expressing flies with neuron-specific *fabp* knockdown (*elavGS*>*GFP-mCherry-Atg8a*, *Aβ42*^2x^, *fabp* i^BL^) or *fabp* overexpression (*elavGS*>*GFP-mCherry-Atg8a*, *Aβ42*^2x^, *fabp*^GX62810^). Flies were grown in medium containing 200 μM RU486 after eclosion and aged for 30 days. Blue dots indicate DAPI-stained nuclei. (B-D) Quantification of the relative ratio of GFP to mCherry puncta (B) and the number of GFP (C) and mCherry (D) puncta in the brains of indicated flies (one-way ANOVA test, *n* = 7, *p < 0.05, **p < 0.01, ***p < 0.001, NS, not significant). (E) Representative confocal images showing GFP-mCherry-Atg8a puncta in the fat bodies of control (*Lsp2*>*GFP-mCherry-Atg8a*/+), *fabp*-knockdown (*Lsp2*>*GFP-mCherry-Atg8a*, *fabp* i^BL^), and *fabp*-overexpression (*Lsp2*>*GFP-mCherry-Atg8a*, *fabp*^GX62810^) flies. (F-H) Quantification of the relative ratio of GFP to mCherry puncta (F) and the number of GFP (G) and mCherry (H) puncta in the third-instar larval fat bodies (*n* ≥ 9, *p < 0.05, **p < 0.01, ***p < 0.001, NS, not significant; F, one-way ANOVA test; G, H, Kruskal-Wallis test). (I) Representative confocal images showing the thioflavin S-stained brains from 20-day-old flies. (J) Quantification of Aβ aggregate intensity in the brains of AD model flies expressing *Atg6* RNAi (*Atg6* i) or *Atg8a* RNAi (*Atg8a* i) with or without *fabp* overexpression (*fabp*^GX62810^) in neurons (one-way ANOVA test, *n* ≥ 8, *p < 0.05, **p < 0.01). (K) Representative confocal images showing apoptotic cells in the brains of 20-day-old flies of the strain indicated. (L) Quantification of the relative number of active Dcp-1-positive cells in the AD model brains expressing *Atg6* i or *Atg8a* i with or without *fabp* overexpression (*fabp*^GX62810^) in neurons (one-way ANOVA test, *n* ≥ 10, *p < 0.05, **p < 0.01). (M) Aversive associative memory performance at 90 s after training of 20-day-old flies of the strain indicated (one-way ANOVA test, *N* = 4, **p < 0.01, ***p < 0.001). (N) Representative confocal images showing apoptotic cells in the brains of *Aβ42*-expressing *fabp*-knockdown flies (*elavGS*>*Aβ42*^2x^, *fabp* i^BL^) fed with (+Rapa) or without (-) 50 μM rapamycin. (O) Quantification of the relative number of apoptotic cells of indicated flies (Student’s *t*-test, *n* = 8, *p < 0.05). (P) Representative confocal images of the wing imaginal discs of third-instar larva immunostained with anti-Atg8a antibody. The right side of each wing disc (where the RFP is expressed in the top left panel) is the posterior region where target genes are regulated by *en-Gal4*. *Atg8a* RNAi was used as a control. (Q, R) *Atg8a* and *Atg6* mRNA levels in the heads of neuron-specific *fabp* knockdown (Q, *elavGS*>*fabp* i^BL^; R, *elavGS*>*fabp* i^KK^) flies with or without 20 μM RU486 treatment (Student’s *t*-test, *p < 0.05, **p < 0.01; Q, *N* = 4; R, *N* = 3). (S) Representative confocal images of the wing imaginal discs of third-instar larva stained with BODIPY. The right side of each wing disc (where the RFP is expressed in the left panels) is the posterior region where target genes are regulated by *en-Gal4*. *Lsd-2* RNAi was used as a control. All flies in (I-O) were grown in medium containing 20 μM RU486 for their entire lives and aged for 20 days. All data are expressed as mean ± SEM. Scale bars: 2 μm (A), 10 μm (E), 50 μm (I, P, S), and 100 μm (K, N). White arrow heads indicate active Dcp-1-positive cells.

Autophagy is critical for Aβ clearance [49]. In our study, *fabp* knockdown increased Aβ accumulation, whereas *fabp* overexpression decreased it (Fig 4A–4D). Therefore, we postulated that fabp may regulate the autophagic process, potentially affecting Aβ clearance. We used the autophagy reporter GFP-mCherry-Atg8a to investigate whether the increased Aβ aggregation induced by *fabp* knockdown was a consequence of autophagic impairment. The absence of green fluorescence in the presence of red fluorescence due to its denaturation in autolysosomes indicates late-stage autophagy. In contrast, the presence of both green and red (yellow) indicates early autophagosomes [50]. When *fabp* was knocked down in neurons lacking Aβ, an increase in yellow puncta was observed in the brain (S5 Fig). This suggests an accumulation of autophagosomes due to the inhibition of basal autophagic processes. In contrast, *fabp* overexpression did not affect basal autophagy levels (S5 Fig), possibly because there was already a sufficient amount of endogenous fabp in the brain to regulate basal autophagy. In parallel with observations in human brain samples [51], *Aβ42* overexpression in the *Drosophila* brain led to the accumulation of autophagosomes due to autophagy impairment (S6 Fig). In the brain of the *Drosophila* AD model with Aβ expression, *fabp* knockdown induced the formation of prominent green puncta, indicating that *fabp* knockdown inhibited the autophagic process (Fig 5A–5D). Conversely, in brains with Aβ expression, unlike in those without Aβ, *fabp* overexpression alleviated Aβ-induced autophagy blockade (Figs 5A–5D and S7). The autophagy-enhancing effect of fabp was also observed in the fat body, the most extensively studied tissue for autophagy in *Drosophila* (Fig 5E–5H). Using the *Lsp2-Gal4* driver, we expressed the GFP-mCherry-Atg8a reporter in the larval fat body and examined the correlation between fabp levels and the extent of autophagy in the fat body. Similar to the brain, *fabp* knockdown induced autophagy blockade, whereas *fabp* overexpression increased autophagy in the fat body (Fig 5E–5H).

To investigate further whether the neuroprotective effect of fabp on Aβ pathology is mediated by enhancing autophagy, we examined the genetic interaction between *fabp* and *Atg6* or *fabp* and *Atg8a*. Expectedly, the knockdown of *Atg6* or *Atg8a* increased Aβ aggregation (Fig 5I and 5J) and Aβ-induced apoptosis (Fig 5K and 5L), whereas autophagy activators such as *mTor*^TED^, a dominant negative form of *mTor*, and *Pi3K59F*, a *Drosophila* ortholog of *Vps34* and a key player in autophagosome formation, decreased them (S8 Fig), indicating that autophagy suppresses Aβ aggregation and Aβ-induced apoptosis. Furthermore, inhibiting autophagy by knocking down *Atg6* or *Atg8a* almost completely abolished the inhibitory effects of *fabp* overexpression on Aβ aggregation and Aβ-induced apoptosis, as well as short-term memory impairment in *Aβ*-expressing flies (Fig 5I–5M), whereas treatment with rapamycin, an autophagy activator, reduced the increased the cell death caused by *fabp* knockdown (Fig 5N and 5O). These findings suggest that fabp may contribute to the protection of neurons from Aβ pathology, possibly through activation of autophagy.

To understand the mechanism of autophagy regulation by fabp, we investigated whether *fabp* knockdown affects autophagy-related gene expression. To this end, we knocked down *fabp* in the posterior region of the wing imaginal disc using the *en-Gal4* driver, and then examined the expression of Atg8a by immunohistochemistry. We found that both of the *fabp* RNAi we used reduced Atg8a expression in a posterior region-specific manner (Fig 5P). We mRNA levels of the autophagy-related genes by RT-qPCR in fly brains with neuron-specific knockdown of *fabp*. The results demonstrated that the expression of *Atg6* and *Atg8a* was diminished by *fabp* RNAi (Fig 5Q and 5R). This indicates that fabp may regulate autophagy by modulating the expression of autophagy-related genes.

Interestingly, we found that knockdown of *Lsd-2*, a gene that plays an important role in lipid storage and metabolism, also reduced Atg8a levels, similar to *fabp* knockdown. This suggests that the accumulation or distribution of lipids may affect autophagy. To provide further evidence in support of this hypothesis, we investigated whether lipid accumulation was altered by *fabp* knockdown. The levels of neutral lipids stained with BODIPY were found to be reduced in the posterior region of the wing discs where *fabp* was knocked down (Fig 5S). The findings of our study suggest that fabp plays a role in autophagy by facilitating the transport of lipids.

### *Drosophila* fabp requires PPAR for autophagy activation

**Fig 6:**
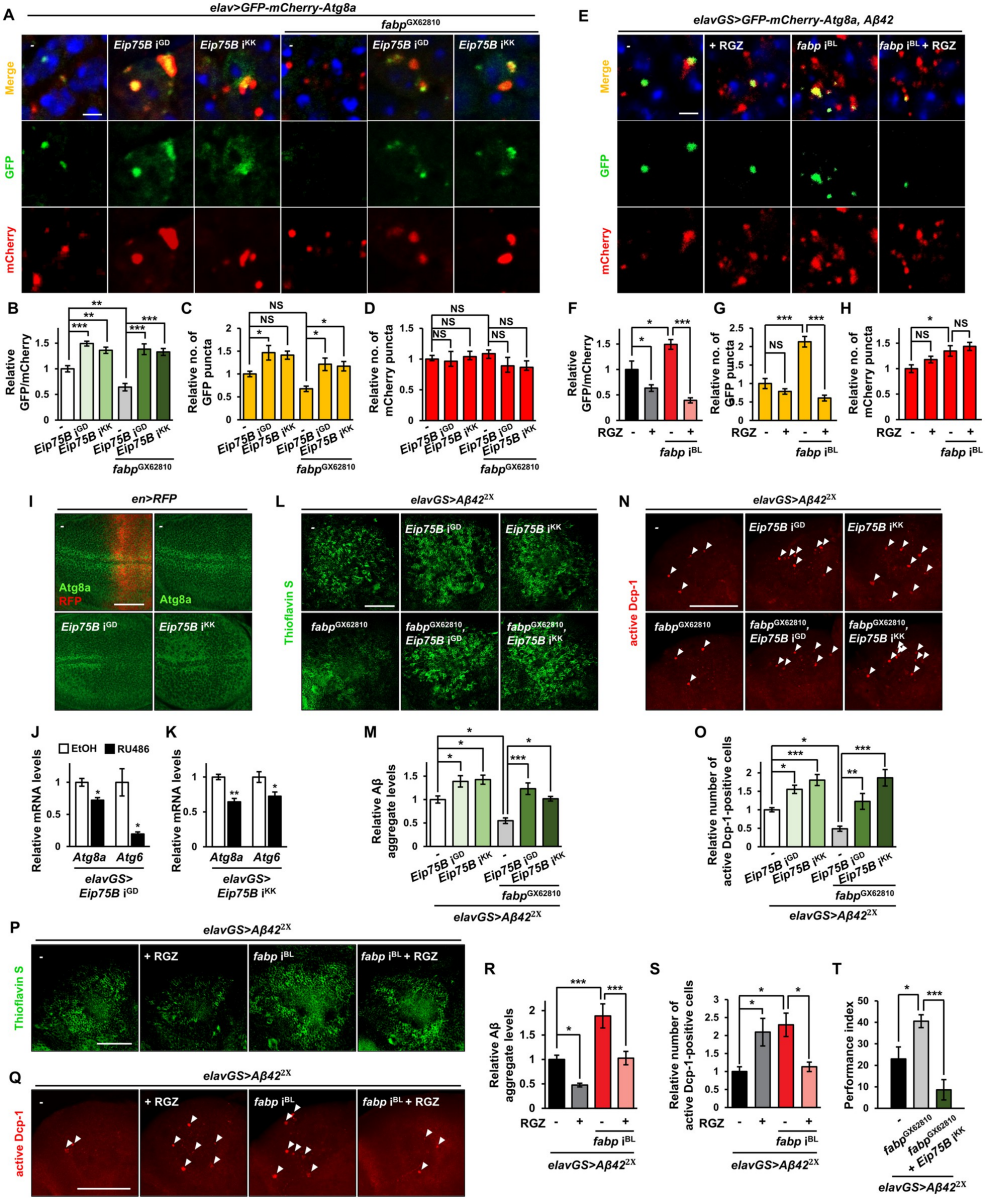
PPAR-dependent autophagy mediates neuroprotective function of fabp. (A) Representative images showing GFP-mCherry-Atg8a puncta in the brains of indicated flies. Blue dots indicate DAPI-stained nuclei. (B-D) Graphs showing the relative ratio of GFP to mCherry puncta (B) and the number of GFP (C) and mCherry (D) puncta in the brains expressing *Eip75B* RNAi (*Eip75B* i^GD^ or *Eip75B* i^KK^) with or without *fabp* overexpression (*fabp*^GX62810^) in neurons (one-way ANOVA test, *n* = 10, *p < 0.05, **p < 0.01, ***p < 0.001, NS, not significant). (E) Representative confocal images showing GFP-mCherry-Atg8a puncta in the brains of control (*elavGS*>*GFP-mCherry-Atg8a*, *Aβ42*^2x^/+) and *fabp*-knockdown (*elavGS*>*GFP-mCherry-Atg8a*, *Aβ42*^2x^, *fabp* i^BL^) flies fed with (+RGZ) or without (-) 50 μM rosiglitazone. (F-H) Graphs showing the relative ratio of GFP to mCherry puncta (F) and the number of GFP (G) and mCherry (H) puncta in the brains of indicated flies (one-way ANOVA test, *n* ≥ 9, *p < 0.05, ***p < 0.001, NS, not significant). (I) Representative confocal images of the wing imaginal discs of third-instar larva immunostained with anti-Atg8a antibody. The right side of each wing disc (where the RFP is expressed in the top left panel) is the posterior region where target genes are regulated by *en-Gal4*. (J, K) *Atg8a* and *Atg6* mRNA levels in the heads of neuron-specific *Eip75B* knockdown (J, *elavGS*>*Eip75B* i^GD^; K, *elavGS*> *Eip75B* i^KK^) flies with or without 20 μM RU486 treatment (*p < 0.05, **p < 0.01; Student’s *t*-test, J, *N* = 3; K, *N* ≥ 3). (L-O) Effect of neuron-specific *Eip75B* knockdown on Aβ aggregation (L, M) and Aβ-induced apoptosis (N, O) in the brains of flies with or without *fabp* overexpression (*fabp*^GX62810^) in neurons. (L, N) Representative confocal images showing the thioflavin S (L)- and active Dcp-1 (N)-stained brains of indicated flies. (M, O) Quantification of Aβ aggregate intensity (M) and the relative number of active Dcp-1-positive cells (O) in the brains of indicated flies (one-way ANOVA test, *p < 0.05, **p < 0.01, ***p < 0.001; M, *n* ≥ 10; O, *n* ≥ 7). (P-S) Effect of 50 μM RGZ treatment on Aβ aggregation (P, R) and Aβ-induced apoptosis (Q, S) in the brains of flies with or without *fabp* RNAi (*fabp* i^BL^) expression in neurons. (P, Q) Representative confocal images showing the thioflavin S (P)- and active Dcp-1 (Q)-stained brains of indicated flies. (R, S) Quantification of Aβ aggregate intensity (R) and the relative number of active Dcp-1-positive cells (S) in the brains of indicated flies (one-way ANOVA test, *p < 0.05, ***p < 0.001; R, *n* ≥ 10; S, *n* ≥ 7). (T) Aversive associative memory performance at 90 s after training of 30-day-old flies of strains indicated (one-way ANOVA test, *N* = 5, *p < 0.05, ***p < 0.001). Flies in (E, P, Q) were grown in medium containing 20 μM RU486 for their entire lives. Due to the unexpected lethal effects observed when *Eip75B* RNAi and *fabp* are expressed simultaneously, flies in (L, N, T) were raised in a medium containing 400 μM RU486 for 30 days, starting immediately after eclosion. This allowed for the expression of these transgenes to be limited to the adult stage and to prevent any potential developmental issues. All data are expressed as mean ± SEM. Scale bars: 2 μm (A, E), 50 μm (I, L, P), and 100 μm (N, Q). White arrow heads indicate active Dcp-1-positive cells.

Given that mammalian Fabp serves as a cytosolic gateway for PPAR ligands and activates PPAR [24], which has been implicated in autophagy [28], we next sought to determine whether autophagy activation by fabp is mediated by PPAR. To address this question, we examined the genetic interaction between *fabp* and *Eip75B*, the only *Drosophila* homolog of PPAR. In the nervous system, similar to *fabp*, *Eip75B* knockdown impaired autophagy (Fig 6A–6D), whereas treatment of rosiglitazone (RGZ), a PPAR activator, promotes it (Fig 6E–6H), suggesting that Eip75B, like mammalian PPAR, also plays an important role in autophagy. Furthermore, *Eip75B* knockdown almost abrogated the autophagy-promoting effect of *fabp* overexpression (Fig 6A–6D), while RGZ rescued the impaired autophagy induced by *fabp* RNAi (Fig 6E–6H). Interestingly, knockdown of *Eip75B* reduced the expression of *Atg6* and *Atg8a* (Fig 6I–6K), as did *fabp*. These results suggest that Eip75B is critically involved in fabp-mediated activation of autophagy. In addition, *Eip75B* knockdown increased Aβ aggregation (Fig 6L and 6M) and Aβ-induced apoptosis (Fig 6N and 6O). RGZ treatment decreased Aβ aggregation, but unexpectedly increased neuronal cell death (Fig 6P–6S), indicating that RGZ has neurotoxicity independent of Aβ. These results suggest that, similarly to fabp, Eip75B plays an important function at least in Aβ clearance. Furthermore, *Eip75B* knockdown almost abolished the inhibitory effect of *fabp* overexpression on Aβ aggregation (Fig 6L and 6M) and Aβ-induced apoptosis (Fig 6N and 6O) and memory impairment (Fig 6T), while RGZ reduced the increased Aβ aggregation (Fig 6P and 6R) and apoptosis (Fig 6Q and 6S) induced by *fabp* knockdown, highlighting the importance of Eip75B in the neuroprotective effect of fabp against Aβ pathology.

### Attenuation of *fabp* knockdown-induced autophagy impairment and exacerbated Aβ pathology following PUFA treatment

**Fig 7:**
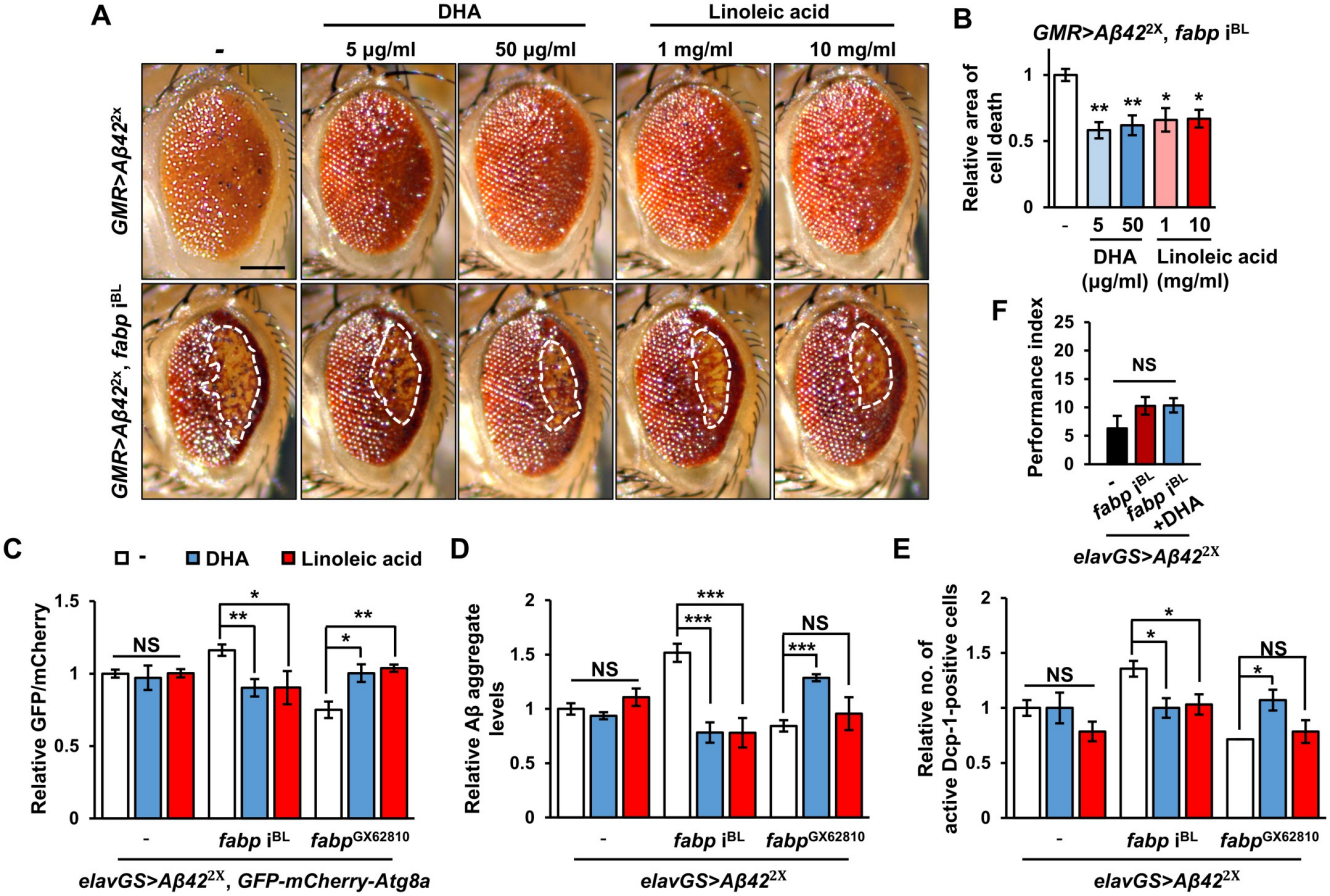
PUFA treatment rescues the enhanced Aβ pathology caused by *fabp* knockdown. (A) The eye phenotypes of *Aβ42*-expressing control (*GMR*>*Aβ42*^2x^/+) and *fabp*-knockdown (*GMR*>*Aβ42*^2x^, *fabp* i^BL^) flies fed with PUFAs (DHA or linoleic acid) at indicated concentrations. The dotted areas in the lower panels indicate the region of cell death. (B) Graph showing the relative cell death area of eye shown in (A) (one-way ANOVA test, *n* ≥ 3, *p < 0.05, **p < 0.01). (C) Relative ratio of GFP to mCherry puncta in control (*elavGS*>*GFP-mCherry-Atg8a*, *Aβ42*^2x^/+), *fabp*-knockdown (*elavGS*>*GFP-mCherry-Atg8a*, *Aβ42*^2x^, *fabp* i^BL^), and *fabp*-overexpression (*elavGS*>*GFP-mCherry-Atg8a*, *Aβ42*^2x^, *fabp*^GX62810^) flies fed with indicated PUFAs (Control, one-way ANOVA test, *n* ≥ 6, NS, not significant; *fabp*-knockdown, one-way ANOVA test, *n* ≥ 4, *p < 0.05, **p < 0.01; *fabp*-overexpression, Kruskal-Wallis test, *n* ≥ 7, *p < 0.05, **p < 0.01). (D) Quantification of Aβ aggregate intensity in thioflavin S-stained brains of control (*elavGS*>*Aβ42*^2x^/+), *fabp*-knockdown (*elavGS*>*Aβ42*^2x^, *fabp* i^BL^), and *fabp*-overexpression (*elavGS*>*Aβ42*^2x^, *fabp*^GX62810^) flies fed with PUFAs (one-way ANOVA test, *n* = 10, ***p < 0.001, NS, not significant). (E) Quantification of the relative number of active Dcp-1-positive cells in the brains of indicated flies fed with PUFAs (Control, Kruskal-Wallis test, *n* = 10, NS, not significant; *fabp*-knockdown, Kruskal-Wallis test, *n* ≥ 9, *p < 0.05; *fabp*-overexpression, one-way ANOVA test, *n* ≥ 8, *p < 0.05, NS, not significant). (F) Aversive associative memory performance at 90 s after training of 20-day-old flies of the strains indicated fed with 50 μg/ml DHA (one-way ANOVA test, *N* = 6, NS, not significant). Flies of all white bar graphs were grown in medium containing ethanol without PUFAs. All flies with *elavGS*-*Gal4* were grown in medium containing 20 μM RU486 for their entire lives and aged for 20 days. All data are expressed as mean ± SEM. Scale bar: 100 μm.

Given the pivotal role of PUFAs as binding partners of fabp, which are also associated with brain function and AD [52], we investigated whether the protective function of fabp against Aβ pathology is related to PUFAs. To address this, we examined the effect of PUFA treatment on the exacerbation of Aβ pathology via knocking down *fabp* expression. Treatment with PUFAs, such as DHA or linoleic acid, had minimal effect on the Aβ-induced rough eye phenotype (Fig 7A, upper panels) but partially attenuated the increased cell death caused by *fabp* knockdown (Fig 7A, lower panels and 7B).

Next, we examined the effect of PUFA treatment on the autophagy blockade induced by *fabp* knockdown in the brain. While PUFA treatment did not affect autophagy in *Aβ42*-expressing brains (Fig 7C), it successfully restored the autophagic flux impaired by *fabp* knockdown to normal levels (Fig 7C). Conversely, under *fabp* overexpression, PUFA treatment suppressed autophagy (Fig 7C). This suggests that appropriate levels of PUFA and fabp are required to regulate autophagy properly. Consistent with autophagy regulation, PUFA treatment of the AD model fly brain with *fabp* knockdown significantly reduced Aβ aggregation and Aβ-induced cell death (Fig 7D and 7E). In contrast, no PUFA effect was observed in those with the normal *fabp* expression (Fig 7D and 7E). However, in brains overexpressing *fabp*, PUFA treatment either increased Aβ aggregation and Aβ-induced cell death or had no effect (Fig 7D and 7E). Despite the neuroprotective effects of DHA in *fabp* knockdown flies, DHA treatment alone was not sufficient to improve memory in *Aβ42*-expressing flies with or without *fabp* knockdown (Fig 7F).

## Discussion

Various *in vitro* and *in vivo* studies have highlighted the role of FABP in neuronal development and function [53–57]. Given the importance of fatty acids in neurons, it is not surprising that FABP, an intraneuronal carrier of fatty acids, plays an important function in neurons. However, due to the diversity of mammalian FABPs and the overlap and complexity of their expression, the *in vivo* function of FABPs in neurons has remained unknown. In contrast, *Drosophila* fabp is encoded by a single gene, and its expression can be easily regulated in specific tissues, making it well-suited for elucidating the role of fabp in these tissues. Building on the advantages of this *Drosophila* system, we previously established a system to tissue-specifically regulate the expression of *fabp* in multiple tissues, including neurons and glial cells. We found that fabp can affect neuronal development and fly behavior in a cell-autonomous or cell-non-autonomous manner [45].

In this study, we manipulated *fabp* expression in neurons and investigated its role in neurons under aging and stress conditions. We found that the suppression of fabp in neurons accelerated age-related proteostasis impairment in the brain, whereas its overexpression inhibited proteostasis impairment during aging. Consistent with these results, *fabp*-knockdown flies showed increased susceptibility to oxidative stress, whereas *fabp*-overexpressing flies were resistant to oxidative stress compared to controls. On the other hand, according to the Aging Fly Cell Atlas [42] and our data, the expression of fabp in the fly head decreases dramatically with age, which may be partially responsible for the impaired proteostasis and increased stress sensitivity of metazoans with age. Interestingly, we also found that modulating neuron-specific *fabp* expression can affect systemic aging. Flies with neuron-specific knockdown or overexpression of *fabp* have a shortened lifespan. However, this is inconsistent with previous reports that global *fabp* overexpression can increase lifespan in the same fly strain we used [43,44]. This may mean that the function of fabp is not only cell autonomous but also cell non-autonomous and may be context-dependent, as seen in previous studies on the role of fabp during development [45].

FABPs are involved in various brain diseases, including AD and PD [1,22,23,53,58]. In particular, FABP3 and FABP7 bind to α-synuclein, a PD-causing factor, leading to α-synuclein oligomerization. Furthermore, inhibition of FABP-α-synuclein interactions via knocking out these FABPs or drug treatment suppresses α-synuclein-induced neurotoxicity [58,59]. On the other hand, little is known about the role of FABP in AD. FABP3 levels are increased in the CSF of patients with AD compared to those in normal individuals, and attempts have been made to use it as a biomarker for AD prediction and progression [60]; however, whether FABP functionally affects the development and progression of AD has not been determined conclusively. *Drosophila* fabp or mammalian Fabp7 rescues *Aβ42*-induced sleep fragmentation [23]. In a mouse model, Fabp7 mediates a neuroprotective pathway through sotilin, a neuronal receptor for apoE. The AD-associated variant apoE4 inhibits sotilin function, leading to Fabp7 degradation [22]. These findings indicate that FABP may play a neuroprotective role in AD, in contrast to PD. Our present results support the hypothesis derived from these previous findings. We have shown that *Drosophila* fabp inhibits Aβ accumulation and reduces Aβ-induced cell death and neurodegeneration, reducing Aβ-induced memory impairment. Therefore, in light of these neuroprotective actions of FABPs, abnormalities in FABP function due to genetic variation may be a risk factor for AD.

In animal cells, proteostasis is impaired with aging, and this impairment is an important marker of aging. This study showed that *fabp* expression decreases with aging in the fly head and that decreased *fabp* expression promotes proteostasis impairment in the aging brain. We also showed that *fabp* expression level affects Aβ accumulation. These results suggest that fabp plays an important role in maintaining proteostasis during aging and under conditions of brain cell stress, such as the presence of Aβ. The decrease in autophagic degradation with age is an important cause of proteostasis impairment [61]. Therefore, as an autophagy regulator, fabp may play important roles in maintaining proteostasis during aging.

Currently, the role of mammalian FABPs in autophagy is controversial [35–39], and autophagy regulation via FABPs may be tissue- or context-dependent. Our results show that *Drosophila* fabp acts as an important autophagy activator, at least in neurons and the fat body, and that the neuroprotective effect of fabp against Aβ neurotoxicity is associated with fabp-induced activation of autophagy. Similarly, *Drosophila* fabp is required to clear photoactivated Rhodopsin-1 protein via endolysosomal pathway [62], suggesting that fabp may be involved in intracellular waste removal in various contexts.

Several FABPs deliver various lipid ligands to their nuclear receptors, PPARs, for latter’s activation [6]. PPARs are sensors of lipid signaling and are involved in various cellular processes, including lipid metabolism, inflammation, and autophagy [28,63]. Given the important functions of FABPs and PPARs in neurons, the FABP-PPAR pathway may be well conserved in the nervous system; however, few studies have examined how they interact in neurons. Here, we found that *Drosophila* fabp plays a crucial role in the accumulation of intracellular lipid droplets that may serve as a source of ligands for PPARs. Moreover, the present study demonstrated that fabp-induced autophagy in neurons was almost abrogated following the knockdown of *Eip75B*, the *Drosophila* homolog of PPAR, and that a PPAR activator rescued the impaired autophagy induced by *fabp* knockdown, suggesting that PPARs mediate fabp-induced autophagy. *Eip75B* knockdown enhanced Aβ-induced cell death and inhibited the neuroprotective effects of fabp, and the PPAR activator reduced the increased Aβ aggregation and apoptosis induced by *fabp* knockdown. Furthermore, knockdown of either *fabp* or *Eip75B* in the wing imaginal disc or adult fly brains resulted in reduced expression of *Atg6* and *Atg8a*, suggesting that fabp regulates autophagy by modulating autophagy-related gene expression. Taken together, these results suggest that the FABP-PPAR pathway is well conserved in *Drosophila* neurons and exerts neuroprotective functions in stressful situations, such as the presence of Aβ.

Consistent with our findings, several previous studies have reported beneficial effects of PPARs in AD and other neurodegenerative diseases [64,65]. A study using a *Drosophila* model of amyotrophic lateral sclerosis showed that PPARγ activation exerts neuroprotective effects [64]. On the other hand, neuroprotection by PPARs can be achieved in various ways, including preventing neuroinflammation, oxidative stress, amyloidogenic pathways, and the activation of autophagy [65]. This suggests that the role of FABP in neurons is likely not limited to autophagic activity, and further studies are needed to understand its function in various cellular processes, including neuroinflammation. In addition, we previously reported that *Drosophila* fabp functions in glial cells as well as neurons [45]. Given the importance of glia in Aβ clearance, the FABP function in glia during aging and in the presence of Aβ should be investigated further.

In conclusion, this study demonstrates that *Drosophila* fabp regulates autophagy via PPAR in neurons, which plays an important role in maintaining proteostasis during aging and stress. It also suggests that the FABP-PPAR pathway is involved in Aβ clearance and may be an important therapeutic target for AD. Further preclinical and clinical studies are required to validate our findings before they may be translated in clinical settings.

## Materials and methods

### *Drosophila* strains

Embryonic lethal abnormal vision (*elav*)-*Gal4* (BL458), *elavGeneSwitch* (*elavGS*)*-Gal4* (BL3642), *w*^1118^ (BL5905), larval serum protein 2 (*Lsp2*)-*Gal4* (BL6357), glass multimer receptor (*GMR*)-*Gal4* (BL9146), *UAS-fabp* RNAi^BL^ (BL34685), *UAS*-Autophagy-related 6 (*Atg6*) RNAi (BL35741), *UAS*-*Atg8a* RNAi (BL80428), *UAS*-*Lsd-2* RNAi (BL34617), *UASp-GFP-mCherry-Atg8a* (BL37749), *UAS-LacZ* (BL8530), *UAS-mTor*^TED^ (BL7013), engrailed (*en*)-*Gal4*, *UAS-RFP* (BL30557), *elav-lexA* (BL52676), and *fabp*^KG06479^ (BL14492) were obtained from the Bloomington *Drosophila* Stock Center (Bloomington, IN, USA). *UAS*-*fabp* RNAi^KK^ (v109169), *UAS*-*Eip75B* RNAi^GD^ (v44851), *UAS*-*Eip75B* RNAi^KK^ (v108399), and *UAS*-*Atg3* RNAi (v101364) were acquired from the Vienna *Drosophila* Stock Center (Vienna, AT). *UAS-Pi3K59F*-3*×HA* (F001151) was acquired from the FlyORF (Zurich, Swiss). *UAS-Aβ42*^2x^, *fabp*^GX62810^, and *LexAop-Aβ42*^Arctic^ were kind gifts from Dr. Pedro Fernandez-Funez (University of Florida, USA), Dr. Kyung-Jin Min (University of Inha, Korea), and Dr. Mark Wu (Johns Hopkins University, USA), respectively.

### Chemicals

The following chemicals were added to the fly medium: RU486 (Sigma, M8046) dissolved in ethanol was added at a concentration of 20 μM, 200 μM, or 400 μM. To test the effect of the PUFAs, DHA (Sigma, D2534) and linoleic acid (Sigma, L1376) were dissolved in ethanol and added to the medium at the concentrations of 5 μg/ml or 50 μg/ml (DHA) and 1 mg/ml or 10 mg/ml (linoleic acid), respectively. The DHA and linoleic acid concentrations were determined based on previous reports [66,67]. Rapamycin (LC Laboratories, R-1000) dissolved in ethanol and rosiglitazone (Sigma, R2408) dissolved in DMSO were added at a concentration of 50 μM each.

### RNA isolation and RT-qPCR

Total RNA was isolated from *Drosophila* heads using TRIzol (Invitrogen, AM9738). For real-time reverse transcription-quantitative polymerase chain reaction (RT-qPCR), cDNA was synthesized using ImProm-II Reverse Transcription System (Promega, A3800), and RT-qPCR was performed using SYBR Green PCR Master Mix (Elpisbio, EBT-1802). Gene expression was quantified using the "delta-delta Ct" method and *RpL32* transcript was utilized for normalization. The following primer pairs were used (forward and reverse): *fabp*, 5′-CACCTCCACCTTCAAGACCT-3′ and 5′-TTAGACGGCCTTGTAGACGC-3′; *sea*, 5′-GCGGCGTTAGAACTACGTG-3′ and 5′-ATGCCCTTAAGGCCCACCT-3′; *Aβ42*, 5′-CACGCCATGGAGGAGTTATT-3′ and 5′-TACTGGTGCAGCTTGATTCG-3′; *Atg8a*, 5′-CAACGTCATTCCACCAACATC-3′ and 5′-CCATGCCGTAAACATTCTCATC-3′; *Atg6*, 5′-TGCACGCAATGGCGGAGTTATCTTTGC-3′ and 5′-CAGCTCCGCTTTCAGCTTAAAAGCAGC-3′; *RpL32*, 5′-AAGCGGCGACGCACTCTGTT-3′ and 5′-GCCCAGCATACAGGCCCAAG-3′.

### Longevity assay

To measure the lifespan of the adult flies, twenty male flies were kept in a vial containing standard cornmeal agar medium at 25°C and 60% humidity. More than five vials (>100 flies) were tested per group. Every three days, the flies were transferred to a new vial and the number of live flies was counted. The experiment was repeated two or three times with independently derived transgenic lines.

### Oxidative stress test

The susceptibility to oxidative stress and its effect on the survival of each genotype was assessed using hydrogen peroxide (H_2_O_2_). Twenty male flies were kept in a vial, and more than five vials (>100 flies) were tested. Briefly, flies of each genotype were starved for 6 h and transferred to agar medium containing 5% sucrose and 1% H_2_O_2_. The number of live flies was counted every 12 h.

### Immunohistochemistry

For immunohistochemical analysis of adult brains, adult flies were fixed for 3 h in 4% paraformaldehyde (PFA) in PBS containing 0.5% TritonX-100 (PBST). After washing with PBST, the brains were dissected, and the samples were incubated with blocking solution [PBST containing 2% bovine serum albumin (BSA) and 5% normal goat serum (NGS)] for 3 h at 25°C. The brains were then incubated with mouse anti-polyubiquitinated protein antibody (Enzo, BML_PW0755-0025 (FK2); 1:200), rabbit anti-Ref(2)P antibody (Abcam, ab178440; 1:200), or mouse anti-β-Amyloid antibody (Santa Cruz, sc-58508, DE2B4; 1:200) for two days at 4°C. After washing with PBST, samples were incubated with Alexa-Fluor-555-conjugated anti-mouse secondary antibody (Invitrogen, A-21424; 1:200) or Alexa-Fluor-488-conjugated anti-rabbit secondary antibody (Invitrogen, A-11034; 1:200) overnight at 4°C. The brains were washed and mounted with Vectashield mounting solution (Vector Laboratories, H-1000) and observed via confocal microscopy (Carl Zeiss, LSM800). Images of Kenyon cell bodies and surrounding brain regions taken at a size of 1,024×1,024 pixels were used to measure the polyubiquitinated protein or Ref(2)P signals. Polyubiquitinated protein or Ref(2)P signals were automatically selected with magic wand tool in Photoshop (Adobe) to exclude the background. Then, the number and the total area of the immunostained dots were measured in the measurement log, and the total area of the dots was divided by the number of dots to get the average value of the dot area (size). Due to the small number and size of polyubiquitinated protein and Ref(2)P signals in the absence of *Aβ42* expression, representative image was cropped to 250×250 pixels to make the dots more visible in the Figs 2B–2D and 2F–2H.

To detect apoptosis, the brains were stained as previously reported [48]. Briefly, the brains were dissected and fixed with 4% PFA for 30 min. After washing, the samples were incubated with blocking solution for 3 h. The brains were incubated with rabbit anti-cleaved *Drosophila* Dcp-1 antibody (Cell signaling, #9578; 1:200) for two days, followed by reaction with Alexa-Fluor-555-conjugated anti-rabbit secondary antibody (Invitrogen, A-21429; 1:200) or Alexa-Fluor-647-conjugated anti-rabbit secondary antibody (Invitrogen, A-21245; 1:200) overnight at 4°C. After washing, the brains were mounted, Kenyon cell bodies and surrounding brain regions were observed.

For immunohistochemistry with the larval wing and eye imaginal discs, larvae were dissected and fixed with 4% PFA for 10 min. The samples were blocked with PBST containing 2% BSA and 2% NGS for 1 h and incubated overnight at 4°C with rabbit anti-Atg8a antibody (Abcam, ab109364; 1:200) or rabbit anti-cleaved *Drosophila* Dcp-1 antibody (Cell signaling, #9578; 1:200), followed by reaction with secondary antibody for 1 h at 25°C.

### Thioflavin S staining

Thioflavin S staining was performed as previously reported [47]. Briefly, adult flies were fixed in 4% PFA containing 0.5% Triton X-100 for 3 h and washed three times with PBST. The brains were dissected and incubated in 50% ethanol containing 0.125% thioflavin S (Sigma-Aldrich, T1892) at 4°C overnight. After washing in 50% ethanol for 10 min, the samples were rinsed with PBST. The brains were mounted with Vectashield (Vector Laboratories, H-1000), Kenyon cell bodies and surrounding brain regions were observed.

### GFP-mCherry-Atg8a puncta measurement

Brains of adult flies were dissected in cold PBS, fixed with 4% PFA for 30 min, and washed three times with PBS. The samples were stained with 1 μg/ml 4′,6-diamidino-2-phenylindole (DAPI) for 10 min. After washing, the brains were mounted with Vectashield (Vector Laboratories, H-1000) and examined via confocal microscopy (Carl Zeiss, LSM800) on the same day. Images taken at a size of 1,024×1,024 pixels were used to count the number of mCherry and GFP puncta, and a representative image was cropped to 115×155 pixels to make the dots more visible in the figure.

For the larval fat bodies, third instar larvae that were not in wondering stage were transferred to a vial containing 1 ml of 20% sucrose solution for 4 h for exposure to starvation conditions. After 4 h, the fat bodies were dissected in PBS, fixed with 4% PFA for 10 min, and washed three times with PBS. Images taken at a size of 1,024×1,024 pixels were used to count the number of mCherry and GFP puncta, and a representative image was cropped to 512×512 pixels to make the dots more visible in the figure.

### Neutral lipid staining

Neutral lipid staining with BODIPY 493/503 was performed as previously reported [68]. Wandering 3^rd^ instar larvae were dissected in PBS and fixed with 4% PFA for 30 min. Tissues were then washed with PBS and stained with PBS containing 2 μg/ml BODIPY 493/503 (Invitrogen, D3922) for 30 min. Following a wash with PBS, the wing imaginal discs were mounted and observed on the same day.

### Histology

The flies were fixed in Carnoy’s solution (60% ethanol, 30% chloroform, and 10% glacial acetic acid) for 3 h at 25°C and fixed in collar for 12 h at 4°C. The specimens were embedded in paraffin. The embedded heads were sectioned (5-μm-thick sections) and stained with hematoxylin and eosin. The stained specimens were examined under a light microscope (Olympus, BX50).

### Western blot analysis

To determine fabp expression in wild-type or *fabp* mutant flies, whole flies were homogenized in 2× Laemmli sample buffer (Bio-Rad, 1610737), and the lysates were separated by SDS-PAGE. Membranes were blocked with 5% BSA and probed with anti-fabp (a gift from Dr. Jason Gerstner; 1:1000) and anti-β-actin (Cell Signaling, #8457; 1:2000) antibodies.

Western blots of detergent-soluble and detergent-insoluble fractions were performed as previously described [69,70]. Adult fly heads were homogenized in Triton X-100 buffer (1% Triton X-100 in PBS containing protease inhibitors) and centrifuged at 4°C for 10 min. The supernatant was collected as a detergent-soluble fraction into a new microcentrifuge tube. The remaining pellet was washed twice with Triton X-100 buffer and centrifuged at 4°C for 5 min. The pellet was resuspended with RIPA buffer containing 8 M urea and 5% SDS, and centrifuged at 4°C for 10 min. The supernatant was collected as a detergent-insoluble fraction. Detergent-soluble and -insoluble fractions were boiled for 5 min after adding 4× Laemmli sample buffer (Bio-Rad, 1610747) containing 2-mercaptoethanol, and separated by SDS-PAGE. Membranes were blocked with 5% BSA and probed with anti-ubiquitin (Cell Signaling, #3936; 1:1000), anti-Ref(2)P (Abcam, ab178440; 1:1000), anti-β-actin (Cell Signaling, #8457; 1:2000), and anti-β-tubulin (DSHB, E7; 1:2000) antibodies.

The primary antibodies were detected using horseradish peroxidase-conjugated secondary antibodies (Cell signaling, #7074, #7076; 1:1000). WesternBright ECL (Advansta, K-12045-D50) reagent was used to visualize the conjugated horseradish peroxidase.

### Measurement of short-term memory

Single-cycle olfactory aversive conditioning was conducted as previously described [71,72]. Briefly, flies were transferred to fresh food vials one day before the experiment. At least 1 h before the experiment, flies were transferred to a darkroom at 25°C and 60% humidity to adapt to the experimental environment. A total of 70~80 flies aged 20 days were placed in a training chamber of the T-maze. Next, 3-Octanol (OCT, Sigma, 218405) or 4-methylcyclohexanol (MCH, Sigma, 153095) was paired with electric shocks (65 V) for 1 min, while the other was not paired. After training, flies were held for 90 s and placed in a test location between two odors for 90 s. A performance index (PI) was calculated to determine that a 50:50 distribution was not learned (PI of 0) and a 0:100 distribution away from the shock-paired odor was perfectly learned (PI of 100). A final performance index was calculated by averaging reciprocal PIs for both odors.

### Statistics

Statistical analysis for comparison between the two groups was performed using Student’s *t*-test. For comparing more than two groups, one-way ANOVA was performed (post-hoc with Tukey-Kramer test for comparing all groups and Dunnett’s test for comparing a control group with multiple experimental groups) if the samples followed a normal distribution, and Kruskal-Wallis test was performed if the samples were not normally distributed. GraphPad Prism (version 8.0) was used to determine statistical significance. For the survival rate analysis, the Online Application for Survival Analysis of Lifespan Assays 2 (https://sbi.postech.ac.kr/oasis2/) was used to determine statistical significance, and the Kaplan–Meier estimator and log-rank test were used to compare the two groups. Significance was defined as *p* < 0.05 (*), *p* < 0.01 (**), or *p* < 0.001 (***).

## Supporting information

S1 Table Lifespan of flies in which *fabp* was knocked down or overexpressed in neurons. *elavGS*/+, control; *elavGS*>*fabp* i^BL^, *fabp* knockdown; *elavGS*>*fabp*^GX62810^, *fabp* overexpression.
(DOCX)

S2 Table Lifespan of flies in which *fabp* was knocked down in neurons. *elavGS*/+, control; *elavGS*>*fabp* i^KK^, *fabp* knockdown.
(DOCX)

S3 Table Lifespan of flies with neuronal *fabp* knockdown induced by *fabp* RNAi^BL^ expression. Flies were grown in ethanol-containing medium without RU486 (−RU486) or 20 μM RU486 (+RU486) for their entire lives.
(DOCX)

S4 Table Lifespan of flies with neuronal *fabp* knockdown induced by *fabp* RNAi^KK^ expression. Flies were grown in ethanol-containing medium without RU486 (−RU486) or 20 μM RU486 (+RU486) for their entire lives.
(DOCX)

S5 Table Lifespan of flies with neuronal *fabp* overexpression induced by RU486 administration. Flies were grown in ethanol-containing medium without RU486 (−RU486) or 20 μM RU486 (+RU486) for their entire lives.
(DOCX)

S6 Table Survival rate of flies with neuron-specific *fabp* knockdown by *fabp* RNAi^BL^ expression under oxidative stress conditions. Flies were grown in 20 μM RU486-containing medium without H_2_O_2_ before eclosion and transferred to 20 μM RU486-containing medium with 1% H_2_O_2_ after eclosion. *elavGS*/+, control; *elavGS*>*fabp* i^BL^, *fabp* knockdown.
(DOCX)

S7 Table Survival rate of flies with neuron-specific *fabp* knockdown by *fabp* RNAi^KK^ expression under oxidative stress conditions. Flies were grown in 20 μM RU486-containing medium without H_2_O_2_ before eclosion and transferred to 20 μM RU486-containing medium with 1% H_2_O_2_ after eclosion. *elavGS*/+, control; *elavGS*>*fabp* i^KK^, *fabp* knockdown.
(DOCX)

S8 Table Survival rate of flies with *fabp* overexpression in neurons under oxidative stress conditions. Flies were grown in 20 μM RU486-containing medium without H_2_O_2_ before eclosion and transferred to 20 μM RU486-containing medium with 1% H_2_O_2_ after eclosion. *elavGS>LacZ*, control; *elavGS>fabp*^GX62810^, *fabp* overexpression.
(DOCX)

S9 Table Lifespan of *Aβ42*-expressing flies with neuron-specific *fabp* knockdown or overexpression. *elavGS*>*Aβ42*^2x^/+, control; *elavGS*>*Aβ42*^2x^, *fabp* i^BL^, *fabp* knockdown; *elavGS*>*Aβ42*^2x^, *fabp*^GX62810^, *fabp* overexpression.
(DOCX)

S10 Table Lifespan of *Aβ42*-expressing flies with neuron-specific *fabp* knockdown. *elavGS*>*Aβ42*^2x^/+, control; *elavGS*>*Aβ42*^2x^, *fabp* i^KK^, *fabp* knockdown.
(DOCX)

S11 Table Lifespan of flies with *Aβ42* expression in neurons. Flies were grown in ethanol-containing medium without RU486 (−RU486) or 20 μM RU486 (+RU486) for their entire lives.
(DOCX)

S12 Table Lifespan of *Aβ42*-expressing flies with neuron-specific *fabp* knockdown by *fabp* RNAi^BL^ expression. Flies were grown in ethanol-containing medium without RU486 (−RU486) or 20 μM RU486 (+RU486) for their entire lives.
(DOCX)

S13 Table Lifespan of *Aβ42*-expressing flies with neuron-specific *fabp* knockdown by *fabp* RNAi^KK^ expression. Flies were grown in ethanol-containing medium without RU486 (−RU486) or 20 μM RU486 (+RU486) for their entire lives.
(DOCX)

S14 Table Survival rate of *Aβ42*-expressing flies with neuron-specific *fabp* knockdown by *fabp* RNAi^BL^ expression under oxidative stress conditions. Flies were grown in 20 μM RU486-containing medium without H_2_O_2_ before eclosion and transferred to 20 μM RU486-containing medium with 1% H_2_O_2_ after eclosion. *elavGS*>*Aβ42*^2x^/+, control; *elavGS*>*Aβ42*^2x^, *fabp* i^BL^, *fabp* knockdown.
(DOCX)

S15 Table Survival rate of *Aβ42*-expressing flies with neuron-specific *fabp* knockdown by *fabp* RNAi^KK^ expression under oxidative stress conditions. Flies were grown in 20 μM RU486-containing medium without H_2_O_2_ before eclosion and transferred to 20 μM RU486-containing medium with 1% H_2_O_2_ after eclosion. *elavGS*>*Aβ42*^2x^/+, control; *elavGS*>*Aβ42*^2x^, *fabp* i^KK^, *fabp* knockdown.
(DOCX)

S16 Table Survival rate of *Aβ42*-expressing flies with neuron-specific *fabp* overexpression under oxidative stress conditions. Flies were grown in 20 μM RU486-containing medium without H_2_O_2_ before eclosion and transferred to 20 μM RU486-containing medium with 1% H_2_O_2_ after eclosion. *elavGS>Aβ42*^2x^, *LacZ*, control; *elavGS>Aβ42*^2x^, *fabp*^GX62810^, *fabp* overexpression.
(DOCX)

S17 Table Raw data for all figure graphs. (XLSX)

S1 Fig Neuron-specific *fabp* knockdown further reduces lifespan in *Aβ42*-expressing flies. Lifespan of *elavGS*>*Aβ42*^2x^/+ (A), *elavGS*>*Aβ42*^2x^, *fabp* i^BL^ (B), and *elavGS*>*Aβ42*^2x^, *fabp* i^KK^ (C) flies with or without 20 μM RU486 treatment for their entire lives [Kaplan–Meier estimator and log-rank test; A, *n* ≥ 81, *p* = 0 (−RU486 vs. +RU486); B, *n* ≥ 105, *p* = 0 (−RU486 vs. +RU486); C, *n* ≥ 105, *p* = 0 (−RU486 vs. +RU486)].
(TIF)

S2 Fig Restoration of short-term memory impairment in *Aβ42*-expressing flies by *fabp* overexpression. Aversive olfactory memory performance at 90 s after training of 20-day-old flies (Student’s *t*-test, *N* = 3, **p < 0.01).
(TIF)

S3 Fig The effect of fabp levels on the *Aβ42*-induced rough-eye phenotype and apoptosis. (A) The eye phenotypes (upper panels) and active Dcp-1-stained eye imaginal discs (lower panels) of control (*GMR*>*Aβ42*^2x^/+), *fabp*-knockdown (*GMR*>*Aβ42*^2x^, *fabp* i^BL^), and *fabp*-overexpression (*GMR*>*Aβ42*^2x^, *fabp*^GX62810^) flies. Yellow arrow heads in the upper panels indicate black spots. (B) Quantification of the relative number of apoptotic cells in the eye imaginal discs (Kruskal-Wallis test, *n* ≥ 10, *p < 0.05). All data are expressed as mean ± SEM. Scale bar: 100 μm.
(TIF)

S4 Fig *Fabp* mutant exacerbates *Aβ42*-induced phenotypes. (A) Western blot analysis showing the reduced fabp protein levels in whole body of *fabp* mutants (*fabp*^-/-^, *fabp*^KG06479^) compared to control (*fabp*^+/+^). (B) Confocal images of the brains from control (*elav*>*Aβ42*, *fabp*^+/+^) and *fabp* heterozygous mutant (*elav*>*Aβ42*, *fabp*^+/-^) flies exhibiting active Dcp-1 immunostaining. (C) Quantification of the relative number of active Dcp-1-positive cells in the brains of indicated flies (Student’s *t*-test, *n* = 14, ***p < 0.001). (D) Confocal images showing the Aβ-stained brains of indicated flies. (E) Quantification of Aβ levels in the indicated brains (Student’s *t*-test, *n* ≥ 16, *p < 0.05). *elav*>*Aβ42*, *elav*-*LexA*>*LexAop*-*Aβ42*^Arc^. All data are expressed as mean ± SEM. Scale bar: 50 μm.
(TIF)

S5 Fig Fabp is required for basal autophagy of neurons. (A) Confocal images showing GFP-mCherry-Atg8a puncta in the brains of control (*elavGS*>*GFP-mCherry-Atg8a*/+), neuronal *fabp*-knockdown (*elavGS*>*GFP-mCherry-Atg8a*, *fabp* i^BL^), and neuronal *fabp*-overexpression (*elavGS*>*GFP-mCherry-Atg8a*, *fabp*^GX62810^) flies. Blue dots indicate DAPI-stained nuclei. (B-D) Quantification of the relative ratio of GFP to mCherry puncta (B) and the number of GFP (C) and mCherry (D) puncta in the brains of indicated flies (*n* ≥ 7, *p < 0.05, **p < 0.01, ***p < 0.001, NS, not significant; B, D, one-way ANOVA test; C, Kruskal-Wallis test). Flies were grown in medium containing 200 μM RU486 after eclosion and aged for 30 days. All data are expressed as mean ± SEM. Scale bar: 2 μm.
(TIF)

S6 Fig Aβ disrupts autophagy in *Drosophila* neurons. (A) Confocal images showing GFP-mCherry-Atg8a puncta in the brains of control (*elav*>*GFP-mCherry-Atg8a*/+) and *Aβ42*- expressing (*elav*>*GFP-mCherry-Atg8a*, *Aβ42*^2x^) flies. Blue dots indicate DAPI-stained nuclei. (B-D) Quantification of the relative ratio of GFP to mCherry puncta (B) and the number of GFP (C) and mCherry (D) puncta in the brain (Student’s *t*-test, *n* ≥ 4, **p < 0.01, ***p < 0.001). All data are expressed as mean ± SEM. Scale bar: 2 μm.
(TIF)

S7 Fig Neuronal fabp increases autophagy flux in AD model. (A) Confocal images showing GFP-mCherry-Atg8a puncta in the brains of control flies (*elavGS*>*GFP-mCherry-Atg8a*, *Aβ42*^2x^/*LacZ*) and *Aβ42*-expressing flies with *fabp* overexpression (*elavGS*>*GFP-mCherry-Atg8a*, *Aβ42*^2x^/*fabp*^GX62810^). (B-D) Quantification of the relative ratio of GFP to mCherry puncta (B) and the number of GFP (C) and mCherry (D) puncta (Student’s *t*-test, *n* ≥ 8, *p < 0.05, NS, not significant). All data are expressed as mean ± SEM. Scale bar: 2 μm.
(TIF)

S8 Fig Overexpression of dominant negative form of mTor or *Pi3K59F* alleviates *Aβ42*-induced phenotypes. (A) Confocal images showing the GFP-mCherry-Atg8a puncta in the brains of flies with overexpression of mTor dominant negative form (*mTor*^TED^). Blue dots indicate DAPI-stained nuclei. (B-D) Quantification of the relative ratio of GFP to mCherry puncta (B), the number of GFP (C), and mCherry (D) puncta in the brains of indicated flies (Student’s *t*-test, *n* ≥ 12, ***p < 0.001). (E-H) Effect of *mTor*^TED^ expression with or without *Atg3* i or *Atg8a* i expression on Aβ aggregation (E, F) and Aβ-induced apoptosis (G, H). (E, G) Confocal images of the brains showing the thioflavin S (E) or active Dcp-1 (G) staining. (F, H) Quantification of the Aβ aggregate intensity (F) or the relative number of active Dcp-1-positive cells (H) in the brains of indicated flies (one-way ANOVA test, *p < 0.05, ***p < 0.001, NS, not significant; F, *n* ≥ 10; H, *n* ≥ 6). (I-L) Effects of *Pi3K59F* expression on Aβ-induced apoptosis (I, J) and neurodegeneration (K, L). (I) Confocal images showing apoptotic cells in the brains of indicated flies. (J) Quantification of the relative number of active Dcp-1-positive cells of indicated flies (Student’s *t*-test, *n* = 10, *p < 0.05). (K) Representative images showing the H&E-stained frontal brain sections of indicated flies. (L) Quantification of the relative area of vacuoles in the brains of indicated flies (Student’s *t*-test, *n* ≥ 16, *p < 0.05). All data are expressed as mean ± SEM. Scale bars: 2 μm (A), 20 μm (K), 50 μm (E), and 100 μm (G, I). White arrow heads indicate active Dcp-1-positive cells and black arrow heads indicate vacuoles.
(TIF)
