# Supplementary material for: Neuronal fatty acid-binding protein enhances autophagy and suppresses amyloid-β pathology in a Drosophila model of Alzheimer’s disease
Source: PLoS Genet. 2024 Nov 19;20(11):e1011475. doi: 10.1371/journal.pgen.1011475 (PMC11575808; doi:10.1371/journal.pgen.1011475)
Supplement: S1 Table — elavGS/+, control; elavGS>fabp iBL, fabp knockdown; elavGS>fabpGX62810, fabp overexpression. (DOCX) [file pgen.1011475.s001.docx]

**S1 Table.** **Lifespan of flies in which *fabp* was knocked down or overexpressed in neurons.**

|  |  |  | Log-rank test | | |
| --- | --- | --- | --- | --- | --- |
|  |  |  | *p*-value | | |
| Strains | No. of flies | Mean lifespan (days) | vs. A | vs. B | vs. C |
| Trial 1 | | | | | |
| *elavGS/+* [A] | 117 | 75.37 ± 1.4 | - | 0 | 0.0214 |
| *elavGS>fabp* i^BL^ [B] | 93 | 55.37 ± 1.93 | 0 | - | 0 |
| *elavGS>fabp*^GX62810^ [C] | 119 | 71.42 ± 1.32 | 0.0214 | 0 | - |
| Trial 2 | | | | | |
| *elavGS/+* [A] | 99 | 75.28 ± 1.58 | - | 0 | 0.0021 |
| *elavGS>fabp* i^BL^ [B] | 120 | 61.58 ± 1.58 | 0 | - | 0.001 |
| *elavGS>fabp*^GX62810^ [C] | 79 | 70.3 ± 1.57 | 0.0021 | 0.001 | - |
| Trial 3 | | | | | |
| *elavGS/+* [A] | 119 | 77.05 ± 1.52 | - | 0 | 5.3e-7 |
| *elavGS>fabp* i^BL^ [B] | 111 | 50.74 ± 1.89 | 0 | - | 0 |
| *elavGS>fabp*^GX62810^ [C] | 108 | 65.73 ± 2.09 | 5.3e-7 | 0 | - |

*elavGS*/+, control; *elavGS*>*fabp* i^BL^, *fabp* knockdown; *elavGS*>*fabp*^GX62810^, *fabp* overexpression.
