# Supplementary material for: Neuronal fatty acid-binding protein enhances autophagy and suppresses amyloid-β pathology in a Drosophila model of Alzheimer’s disease
Source: PLoS Genet. 2024 Nov 19;20(11):e1011475. doi: 10.1371/journal.pgen.1011475 (PMC11575808; doi:10.1371/journal.pgen.1011475)
Supplement: S2 Table — elavGS/+, control; elavGS>fabp iKK, fabp knockdown. (DOCX) [file pgen.1011475.s002.docx]

**S2 Table. Lifespan of flies in which *fabp* was knocked down in neurons.**

|  |  |  | Log-rank test | |
| --- | --- | --- | --- | --- |
|  |  |  | *p*-value | |
| Strains | No. of flies | Mean lifespan (days) | vs. A | vs. B |
| Trial 1 | | | | |
| *elavGS/+* [A] | 117 | 55.09 ± 1.66 | - | 0.0021 |
| *elavGS>fabp* i^KK^ [B] | 119 | 51.03 ± 1.36 | 0.0021 | - |
| Trial 2 | | | | |
| *elavGS/+* [A] | 117 | 54.34 ± 1.47 | - | 0.001 |
| *elavGS>fabp* i^KK^ [B] | 119 | 46.7 ± 1.46 | 0.001 | - |
| Trial 3 | | | | |
| *elavGS/+* [A] | 109 | 49.66 ± 1.45 | - | 0.0000023 |
| *elavGS>fabp* i^KK^ [B] | 100 | 41.07 ± 1.33 | 0.0000023 | - |

*elavGS*/+, control; *elavGS*>*fabp* i^KK^, *fabp* knockdown.
