# Supplementary material for: Neuronal fatty acid-binding protein enhances autophagy and suppresses amyloid-β pathology in a Drosophila model of Alzheimer’s disease
Source: PLoS Genet. 2024 Nov 19;20(11):e1011475. doi: 10.1371/journal.pgen.1011475 (PMC11575808; doi:10.1371/journal.pgen.1011475)
Supplement: S3 Table — Flies were grown in ethanol-containing medium without RU486 (−RU486) or 20 μM RU486 (+RU486) for their entire lives. (DOCX) [file pgen.1011475.s003.docx]

**S3 Table.** **Lifespan of flies with neuronal *fabp* knockdown induced by *fabp* RNAi^BL^ expression.**

|  |  |  | Log-rank test | |
| --- | --- | --- | --- | --- |
|  |  |  | *p*-value | |
| Strain: *elavGS*>*fabp* i^BL^ | No. of flies | Mean lifespan (days) | vs. A | vs. B |
| Trial 1 | | | | |
| - RU486 [A] | 112 | 65.1 ± 0.95 | - | 0 |
| + RU486 [B] | 113 | 45.14 ± 1.65 | 0 | - |
| Trial 2 | | | | |
| - RU486 [A] | 117 | 62.59 ± 1.04 | - | 0 |
| + RU486 [B] | 113 | 38.31 ± 1.51 | 0 | - |
| Trial 3 | | | | |
| - RU486 [A] | 114 | 68.67 ± 1.03 | - | 0 |
| + RU486 [B] | 116 | 41.47 ± 1.59 | 0 | - |

Flies were grown in ethanol-containing medium without RU486 (−RU486) or 20 μM RU486 (+RU486) for their entire lives.
