# Supplementary material for: Neuronal fatty acid-binding protein enhances autophagy and suppresses amyloid-β pathology in a Drosophila model of Alzheimer’s disease
Source: PLoS Genet. 2024 Nov 19;20(11):e1011475. doi: 10.1371/journal.pgen.1011475 (PMC11575808; doi:10.1371/journal.pgen.1011475)
Supplement: S5 Table — Flies were grown in ethanol-containing medium without RU486 (−RU486) or 20 μM RU486 (+RU486) for their entire lives. (DOCX) [file pgen.1011475.s005.docx]

**S5 Table.** **Lifespan of flies with neuronal *fabp* overexpression induced by RU486 administration.**

|  |  |  | Log-rank test | |
| --- | --- | --- | --- | --- |
|  |  |  | *p*-value | |
| Strain: *elavGS*>*fabp*^GX62810^ | No. of flies | Mean lifespan (days) | vs. A | vs. B |
| Trial 1 | | | | |
| - RU486 [A] | 113 | 64.99 ± 1.76 | - | 0.0009 |
| + RU486 [B] | 108 | 59.83 ± 1.66 | 0.0009 | - |
| Trial 2 | | | | |
| - RU486 [A] | 110 | 55.38 ± 1.92 | - | 0.0018 |
| + RU486 [B] | 86 | 52.31 ± 1.67 | 0.0018 | - |
| Trial 3 | | | | |
| - RU486 [A] | 108 | 60.91 ± 1.49 | - | 0.000029 |
| + RU486 [B] | 116 | 56.20 ± 1.17 | 0.000029 | - |

Flies were grown in ethanol-containing medium without RU486 (−RU486) or 20 μM RU486 (+RU486) for their entire lives.
