# Supplementary material for: Neuronal fatty acid-binding protein enhances autophagy and suppresses amyloid-β pathology in a Drosophila model of Alzheimer’s disease
Source: PLoS Genet. 2024 Nov 19;20(11):e1011475. doi: 10.1371/journal.pgen.1011475 (PMC11575808; doi:10.1371/journal.pgen.1011475)
Supplement: S6 Table — Flies were grown in 20 μM RU486-containing medium without H2O2 before eclosion and transferred to 20 μM RU486-containing medium with 1% H2O2 after eclosion. elavGS/+, control; elavGS>fabp iBL, fabp knockdown. (DOCX) [file pgen.1011475.s006.docx]

**S6 Table. Survival rate of flies with neuron-specific *fabp* knockdown by *fabp* RNAi^BL^ expression under oxidative stress conditions.**

|  |  |  | Log-rank test | |
| --- | --- | --- | --- | --- |
|  |  |  | *p*-value | |
| Strains | No. of flies | Mean lifespan (hours) | vs. A | vs. B |
| Trial 1 | | | | |
| *elavGS/+* [A] | 116 | 87.83 ± 1.68 | - | 0.0006 |
| *elavGS>fabp* i^BL^ [B] | 98 | 79.47 ± 1.53 | 0.0006 | - |
| Trial 2 | | | | |
| *elavGS/+* [A] | 119 | 90.76 ± 2.19 | - | 0 |
| *elavGS>fabp* i^BL^ [B] | 97 | 71.63 ± 1.29 | 0 | - |

Flies were grown in 20 µM RU486-containing medium without H_2_O_2_ before eclosion and transferred to 20 µM RU486-containing medium with 1% H_2_O_2_ after eclosion. *elavGS*/+, control; *elavGS*>*fabp* i^BL^, *fabp* knockdown.
