# Supplementary material for: Neuronal fatty acid-binding protein enhances autophagy and suppresses amyloid-β pathology in a Drosophila model of Alzheimer’s disease
Source: PLoS Genet. 2024 Nov 19;20(11):e1011475. doi: 10.1371/journal.pgen.1011475 (PMC11575808; doi:10.1371/journal.pgen.1011475)
Supplement: S9 Table — elavGS>Aβ422x/+, control; elavGS>Aβ422x, fabp iBL, fabp knockdown; elavGS>Aβ422x, fabpGX62810, fabp overexpression. (DOCX) [file pgen.1011475.s009.docx]

**S9 Table. Lifespan of *Aβ42*-expressing flies with neuron-specific *fabp* knockdown or overexpression.**

|  |  |  | Log-rank test | | |
| --- | --- | --- | --- | --- | --- |
|  |  |  | *p*-value | | |
| Strains | No. of flies | Mean lifespan (days) | vs. A | vs. B | vs. C |
| Trial 1 | | | | | |
| *elavGS>Aβ42*^2x^*/+* [A] | 118 | 67.95 ± 1.24 | - | 0 | 0.7601 |
| *elavGS>Aβ42*^2x^*, fabp* i^BL^ [B] | 119 | 45.63 ± 1.6 | 0 | - | 0 |
| *elavGS>Aβ42*^2x^*, fabp*^GX62810^ [C] | 117 | 68.68 ± 1.21 | 0.7601 | 0 | - |
| Trial 2 | | | | | |
| *elavGS>Aβ42*^2x^*/+* [A] | 112 | 73.84 ± 1.58 | - | 0 | 0.5076 |
| *elavGS>Aβ42*^2x^*, fabp* i^BL^ [B] | 98 | 54.86 ± 1.93 | 0 | - | 0 |
| *elavGS>Aβ42*^2x^*, fabp*^GX62810^ [C] | 98 | 74.37 ± 1.23 | 0.5076 | 0 | - |
| Trial 3 | | | | | |
| *elavGS>Aβ42*^2x^*/+* [A] | 91 | 63.97 ± 2.36 | - | 0 | 0.9056 |
| *elavGS>Aβ42*^2x^*, fabp* i^BL^ [B] | 64 | 41.41 ± 2.06 | 0 | - | 0 |
| *elavGS>Aβ42*^2x^*, fabp*^GX62810^ [C] | 84 | 64.26 ± 2.39 | 0.9056 | 0 | - |

*elavGS*>*Aβ42*^2x^/+, control; *elavGS*>*Aβ42*^2x^, *fabp* i^BL^, *fabp* knockdown; *elavGS*>*Aβ42*^2x^, *fabp*^GX62810^, *fabp* overexpression.
