# Supplementary material for: Neuronal fatty acid-binding protein enhances autophagy and suppresses amyloid-β pathology in a Drosophila model of Alzheimer’s disease
Source: PLoS Genet. 2024 Nov 19;20(11):e1011475. doi: 10.1371/journal.pgen.1011475 (PMC11575808; doi:10.1371/journal.pgen.1011475)
Supplement: S10 Table — elavGS>Aβ422x/+, control; elavGS>Aβ422x, fabp iKK, fabp knockdown. (DOCX) [file pgen.1011475.s010.docx]

**S10 Table. Lifespan of *Aβ42*-expressing flies with neuron-specific *fabp* knockdown.**

|  |  |  | Log-rank test | |
| --- | --- | --- | --- | --- |
|  |  |  | *p*-value | |
| Strains | No. of flies | Mean lifespan (days) | vs. A | vs. B |
| Trial 1 | | | | |
| *elavGS>Aβ42*^2x^*/+* [A] | 119 | 58.42 ± 1.03 | - | 1.2e-7 |
| *elavGS>Aβ42*^2x^*, fabp* i^KK^ [B] | 115 | 50.57 ± 1.26 | 1.2e-7 | - |
| Trial 2 | | | | |
| *elavGS>Aβ42*^2x^*/+* [A] | 112 | 55.44 ± 1.65 | - | 0.000014 |
| *elavGS>Aβ42*^2x^*, fabp* i^KK^ [B] | 117 | 48.21 ± 1.4 | 0.000014 | - |

*elavGS*>*Aβ42*^2x^/+, control; *elavGS*>*Aβ42*^2x^, *fabp* i^KK^, *fabp* knockdown.
