# Supplementary material for: Neuronal fatty acid-binding protein enhances autophagy and suppresses amyloid-β pathology in a Drosophila model of Alzheimer’s disease
Source: PLoS Genet. 2024 Nov 19;20(11):e1011475. doi: 10.1371/journal.pgen.1011475 (PMC11575808; doi:10.1371/journal.pgen.1011475)
Supplement: S11 Table — Flies were grown in ethanol-containing medium without RU486 (−RU486) or 20 μM RU486 (+RU486) for their entire lives. (DOCX) [file pgen.1011475.s011.docx]

**S11 Table.** **Lifespan of flies with *Aβ42* expression in neurons.**

|  |  |  | Log-rank test | |
| --- | --- | --- | --- | --- |
|  |  |  | *p*-value | |
| Strain: *elavGS*>*Aβ42*^2x^*/+* | No. of flies | Mean lifespan (days) | vs. A | vs. B |
| Trial 1 | | | | |
| - RU486 [A] | 81 | 54.05 ± 0.79 | - | 0 |
| + RU486 [B] | 106 | 41.09 ± 0.92 | 0 | - |
| Trial 2 | | | | |
| - RU486 [A] | 92 | 58.95 ± 0.71 | - | 0 |
| + RU486 [B] | 108 | 45.81 ± 1.14 | 0 | - |
| Trial 3 | | | | |
| - RU486 [A] | 98 | 50.47 ± 0.91 | - | 0 |
| + RU486 [B] | 107 | 41.84 ± 1.06 | 0 | - |

Flies were grown in ethanol-containing medium without RU486 (−RU486) or 20 μM RU486 (+RU486) for their entire lives.
