# Supplementary material for: Neuronal fatty acid-binding protein enhances autophagy and suppresses amyloid-β pathology in a Drosophila model of Alzheimer’s disease
Source: PLoS Genet. 2024 Nov 19;20(11):e1011475. doi: 10.1371/journal.pgen.1011475 (PMC11575808; doi:10.1371/journal.pgen.1011475)
Supplement: S13 Table — Flies were grown in ethanol-containing medium without RU486 (−RU486) or 20 μM RU486 (+RU486) for their entire lives. (DOCX) [file pgen.1011475.s013.docx]

**S13 Table.** **Lifespan of *Aβ42*-expressing flies with neuron-specific *fabp* knockdown by *fabp* RNAi^KK^ expression.**

|  |  |  | Log-rank test | |
| --- | --- | --- | --- | --- |
|  |  |  | *p*-value | |
| Strain: *elavGS*>*Aβ42*^2x^, *fabp* i^KK^ | No. of flies | Mean lifespan (days) | vs. A | vs. B |
| Trial 1 | | | | |
| - RU486 [A] | 105 | 58.49 ± 0.95 | - | 0 |
| + RU486 [B] | 106 | 41.27 ± 1.15 | 0 | - |
| Trial 2 | | | | |
| - RU486 [A] | 108 | 57.13 ± 0.59 | - | 0 |
| + RU486 [B] | 105 | 39.27 ± 1.17 | 0 | - |

Flies were grown in ethanol-containing medium without RU486 (−RU486) or 20 μM RU486 (+RU486) for their entire lives.
