# Supplementary material for: Neuronal fatty acid-binding protein enhances autophagy and suppresses amyloid-β pathology in a Drosophila model of Alzheimer’s disease
Source: PLoS Genet. 2024 Nov 19;20(11):e1011475. doi: 10.1371/journal.pgen.1011475 (PMC11575808; doi:10.1371/journal.pgen.1011475)
Supplement: S15 Table — Flies were grown in 20 μM RU486-containing medium without H2O2 before eclosion and transferred to 20 μM RU486-containing medium with 1% H2O2 after eclosion. elavGS>Aβ422x/+, control; elavGS>Aβ422x, fabp iKK, fabp knockdown. (DOCX) [file pgen.1011475.s015.docx]

**S15 Table. Survival rate of *Aβ42*-expressing flies with neuron-specific *fabp* knockdown by *fabp* RNAi^KK^ expression under oxidative stress conditions.**

|  |  |  | Log-rank test | |
| --- | --- | --- | --- | --- |
|  |  |  | *p*-value | |
| Strains | No. of flies | Mean lifespan (hours) | vs. A | vs. B |
| Trial 1 | | | | |
| *elavGS>Aβ42*^2x^*/+* [A] | 120 | 116 ± 2.05 | - | 0 |
| *elavGS>Aβ42*^2x^*, fabp* i^KK^ [B] | 86 | 96.56 ± 1.63 | 0 | - |
| Trial 2 | | | | |
| *elavGS>Aβ42*^2x^*/+* [A] | 113 | 111.29 ± 1.8 | - | 0.0000044 |
| *elavGS>Aβ42*^2x^*, fabp* i^KK^ [B] | 97 | 97.98 ± 1.86 | 0.0000044 | - |
| Trial 3 | | | | |
| *elavGS>Aβ42*^2x^*/+* [A] | 118 | 116.03 ± 1.89 | - | 0 |
| *elavGS>Aβ42*^2x^*, fabp* i^KK^ [B] | 94 | 94.21 ± 1.68 | 0 | - |

Flies were grown in 20 µM RU486-containing medium without H_2_O_2_ before eclosion and transferred to 20 µM RU486-containing medium with 1% H_2_O_2_ after eclosion. *elavGS*>*Aβ42*^2x^/+, control; *elavGS*>*Aβ42*^2x^, *fabp* i^KK^, *fabp* knockdown.
