# Supplementary material for: Neuronal fatty acid-binding protein enhances autophagy and suppresses amyloid-β pathology in a Drosophila model of Alzheimer’s disease
Source: PLoS Genet. 2024 Nov 19;20(11):e1011475. doi: 10.1371/journal.pgen.1011475 (PMC11575808; doi:10.1371/journal.pgen.1011475)
Supplement: S16 Table — Flies were grown in 20 μM RU486-containing medium without H2O2 before eclosion and transferred to 20 μM RU486-containing medium with 1% H2O2 after eclosion. elavGS>Aβ422x, LacZ, control; elavGS>Aβ422x, fabpGX62810, fabp overexpression. (DOCX) [file pgen.1011475.s016.docx]

**S16 Table. Survival rate of *Aβ42*-expressing flies with neuron-specific *fabp* overexpression under oxidative stress conditions.**

|  |  |  | Log-rank test | |
| --- | --- | --- | --- | --- |
|  |  |  | *p*-value | |
| Strains | No. of flies | Mean lifespan (hours) | vs. A | vs. B |
| Trial 1 | | | | |
| *elavGS>Aβ42*^2x^*, LacZ* [A] | 120 | 104.5 ± 2.26 | - | 0.0000073 |
| *elavGS>Aβ42*^2x^*, fabp*^GX62810^ [B] | 120 | 119.7 ± 2.35 | 0.0000073 | - |
| Trial 2 | | | | |
| *elavGS>Aβ42*^2x^*, LacZ* [A] | 120 | 104.1 ± 3.42 | - | 0.0007 |
| *elavGS>Aβ42*^2x^*, fabp*^GX62810^ [B] | 120 | 122.4 ± 2.75 | 0.0007 | - |

Flies were grown in 20 µM RU486-containing medium without H_2_O_2_ before eclosion and transferred to 20 µM RU486-containing medium with 1% H_2_O_2_ after eclosion. *elavGS*>*Aβ42*^2x^*, LacZ*, control; *elavGS*>*Aβ42*^2x^, *fabp*^GX62810^, *fabp* overexpression.
