# Supplementary figures and images for: Neuronal fatty acid-binding protein enhances autophagy and suppresses amyloid-β pathology in a Drosophila model of Alzheimer’s disease
Source: PLoS Genet. 2024 Nov 19;20(11):e1011475. doi: 10.1371/journal.pgen.1011475 (PMC11575808; doi:10.1371/journal.pgen.1011475)

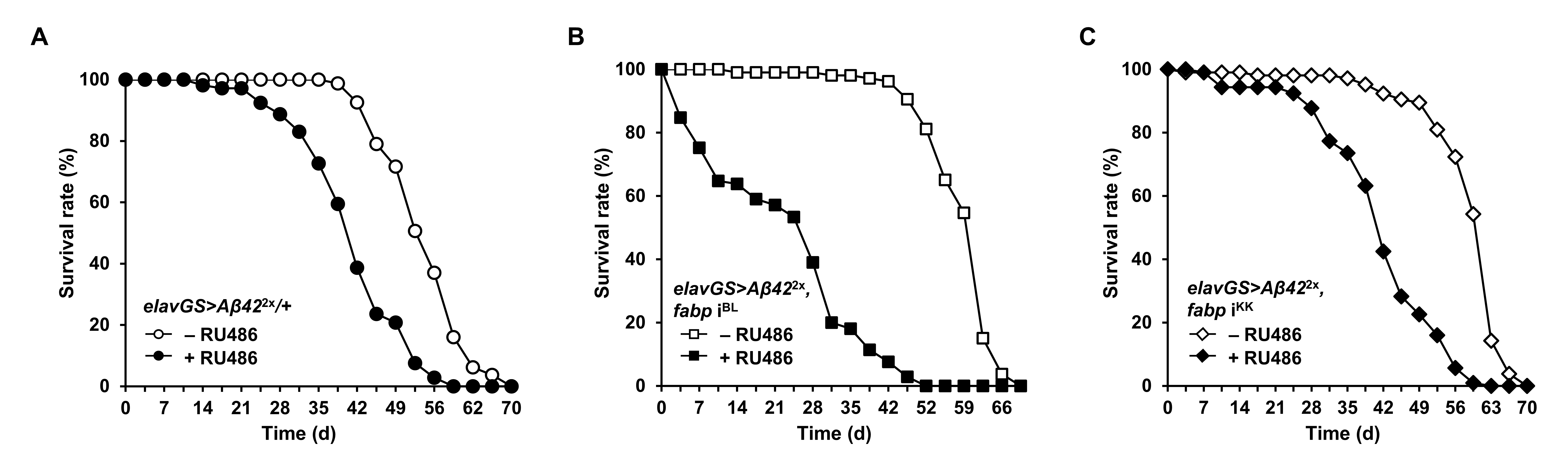

Supplement: S1 Fig — Lifespan of elavGS>Aβ422x/+ (A), elavGS>Aβ422x, fabp iBL (B), and elavGS>Aβ422x, fabp iKK (C) flies with or without 20 μM RU486 treatment for their entire lives [Kaplan–Meier estimator and log-rank test; A, n ≥ 81, p = 0 (−RU486 vs. +RU486); B, n ≥ 105, p = 0 (−RU486 vs. +RU486); C, n ≥ 105, p = 0 (−RU486 vs. +RU486)]. (TIF) [file pgen.1011475.s018.TIF]

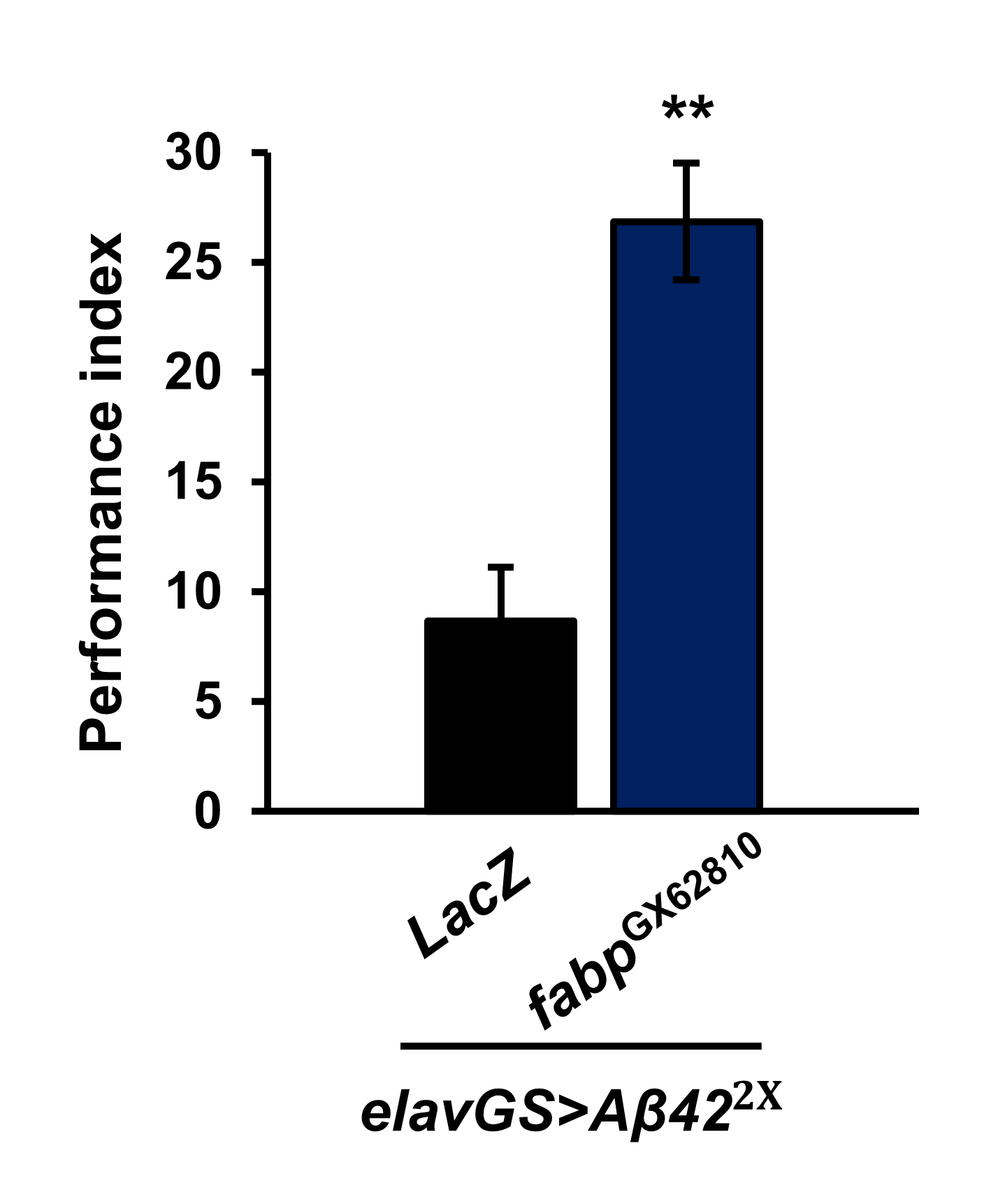

Supplement: S2 Fig — Aversive olfactory memory performance at 90 s after training of 20-day-old flies (Student’s t-test, N = 3, **p < 0.01). (TIF) [file pgen.1011475.s019.TIF]

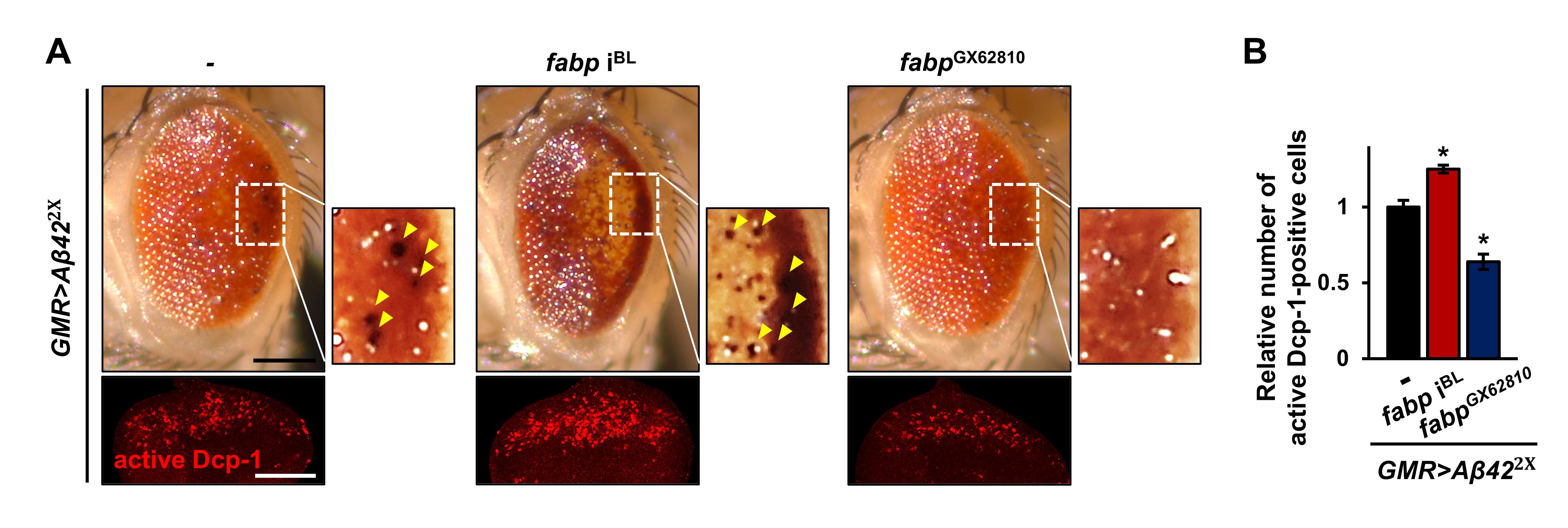

Supplement: S3 Fig — (A) The eye phenotypes (upper panels) and active Dcp-1-stained eye imaginal discs (lower panels) of control (GMR>Aβ422x/+), fabp-knockdown (GMR>Aβ422x, fabp iBL), and fabp-overexpression (GMR>Aβ422x, fabpGX62810) flies. Yellow arrow heads in the upper panels indicate black spots. (B) Quantification of the relative number of apoptotic cells in the eye imaginal discs (Kruskal-Wallis test, n ≥ 10, *p < 0.05). All data are expressed as mean ± SEM. Scale bar: 100 μm. (TIF) [file pgen.1011475.s020.TIF]

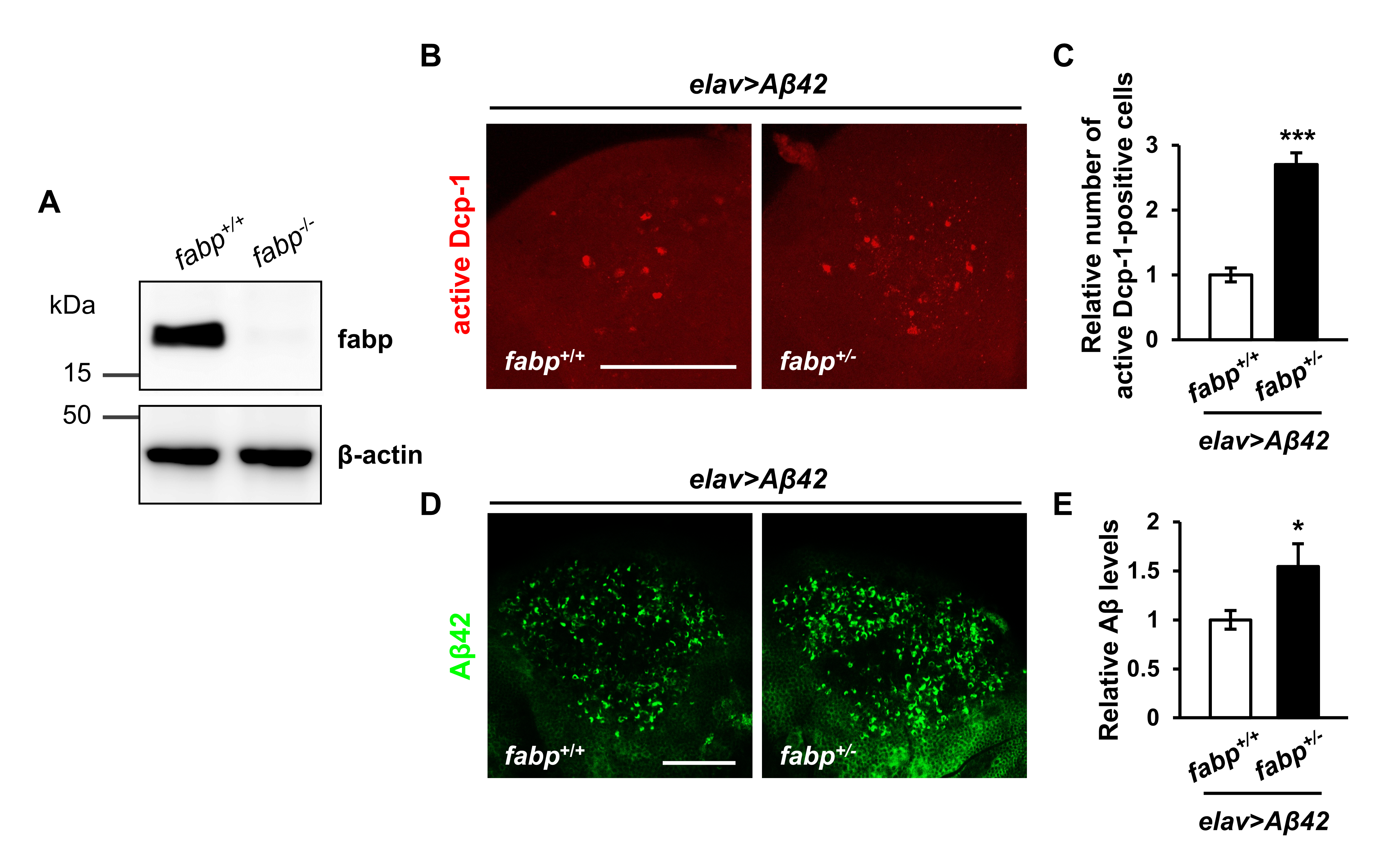

Supplement: S4 Fig — (A) Western blot analysis showing the reduced fabp protein levels in whole body of fabp mutants (fabp-/-, fabpKG06479) compared to control (fabp+/+). (B) Confocal images of the brains from control (elav>Aβ42, fabp+/+) and fabp heterozygous mutant (elav>Aβ42, fabp+/-) flies exhibiting active Dcp-1 immunostaining. (C) Quantification of the relative number of active Dcp-1-positive cells in the brains of indicated flies (Student’s t-test, n = 14, ***p < 0.001). (D) Confocal images showing the Aβ-stained brains of indicated flies. (E) Quantification of Aβ levels in the indicated brains (Student’s t-test, n ≥ 16, *p < 0.05). elav>Aβ42, elav-LexA>LexAop-Aβ42Arc. All data are expressed as mean ± SEM. Scale bar: 50 μm. (TIF) [file pgen.1011475.s021.TIF]

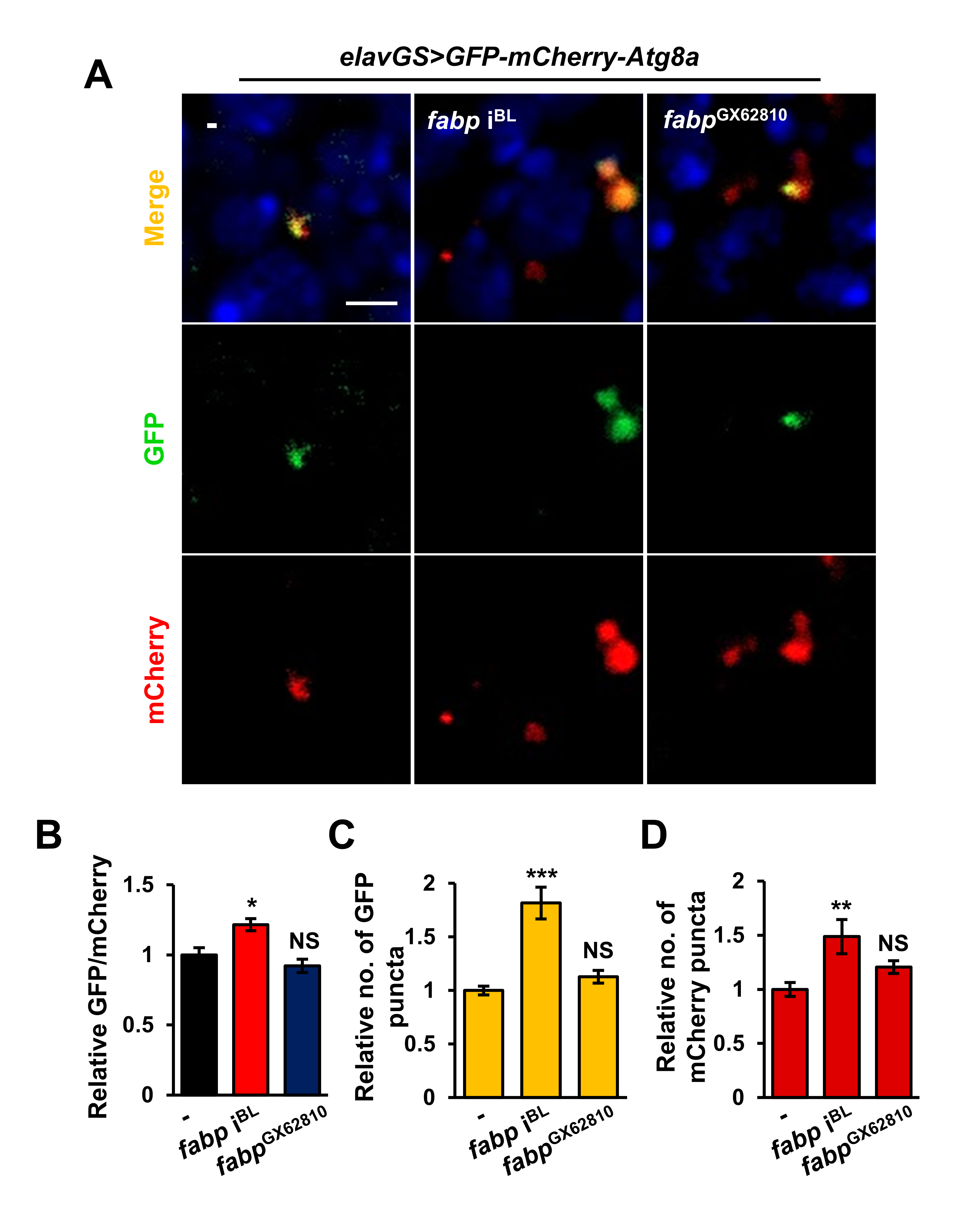

Supplement: S5 Fig — (A) Confocal images showing GFP-mCherry-Atg8a puncta in the brains of control (elavGS>GFP-mCherry-Atg8a/+), neuronal fabp-knockdown (elavGS>GFP-mCherry-Atg8a, fabp iBL), and neuronal fabp-overexpression (elavGS>GFP-mCherry-Atg8a, fabpGX62810) flies. Blue dots indicate DAPI-stained nuclei. (B-D) Quantification of the relative ratio of GFP to mCherry puncta (B) and the number of GFP (C) and mCherry (D) puncta in the brains of indicated flies (n ≥ 7, *p < 0.05, **p < 0.01, ***p < 0.001, NS, not significant; B, D, one-way ANOVA test; C, Kruskal-Wallis test). Flies were grown in medium containing 200 μM RU486 after eclosion and aged for 30 days. All data are expressed as mean ± SEM. Scale bar: 2 μm. (TIF) [file pgen.1011475.s022.TIF]

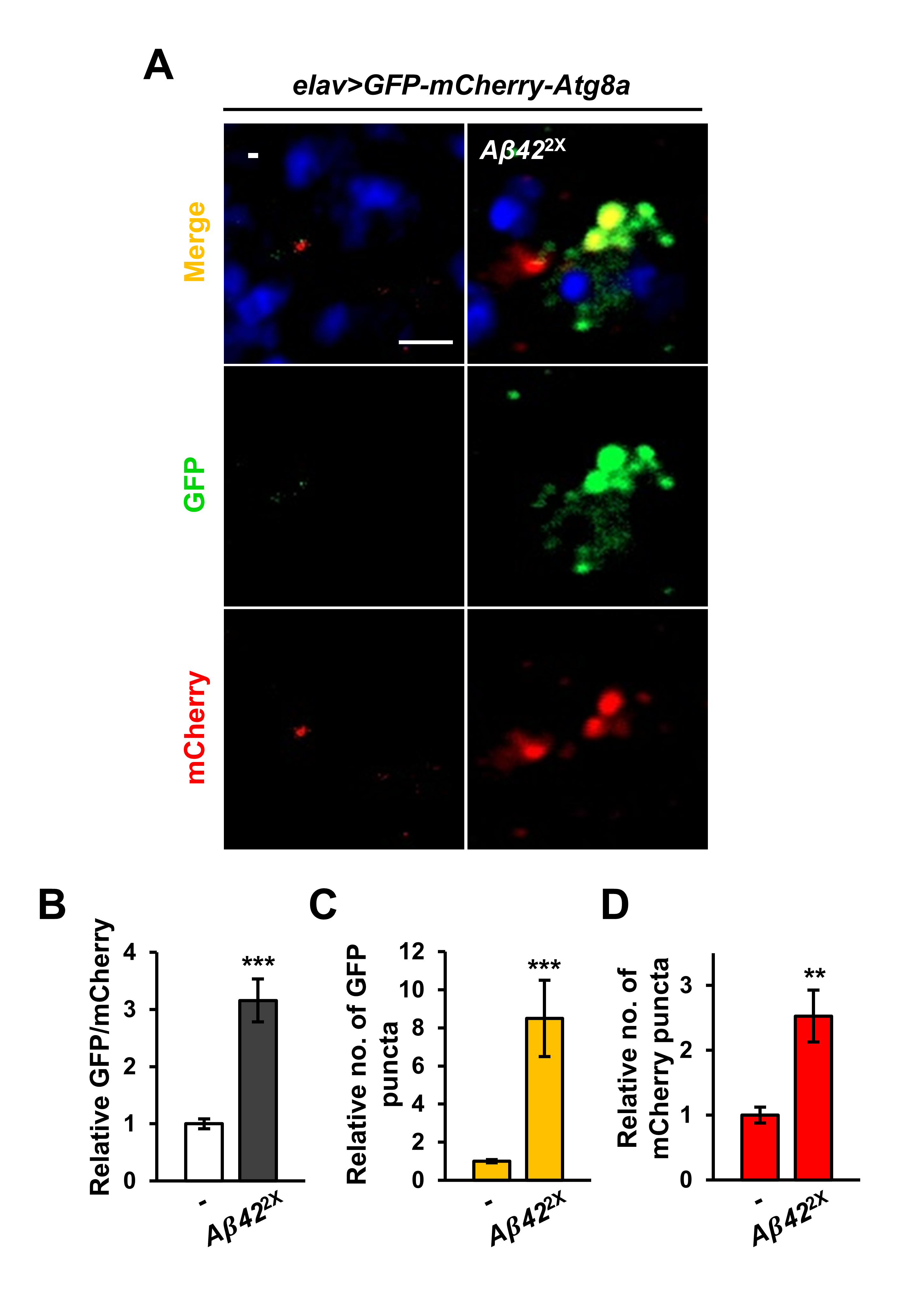

Supplement: S6 Fig — (A) Confocal images showing GFP-mCherry-Atg8a puncta in the brains of control (elav>GFP-mCherry-Atg8a/+) and Aβ42- expressing (elav>GFP-mCherry-Atg8a, Aβ422x) flies. Blue dots indicate DAPI-stained nuclei. (B-D) Quantification of the relative ratio of GFP to mCherry puncta (B) and the number of GFP (C) and mCherry (D) puncta in the brain (Student’s t-test, n ≥ 4, **p < 0.01, ***p < 0.001). All data are expressed as mean ± SEM. Scale bar: 2 μm. (TIF) [file pgen.1011475.s023.TIF]

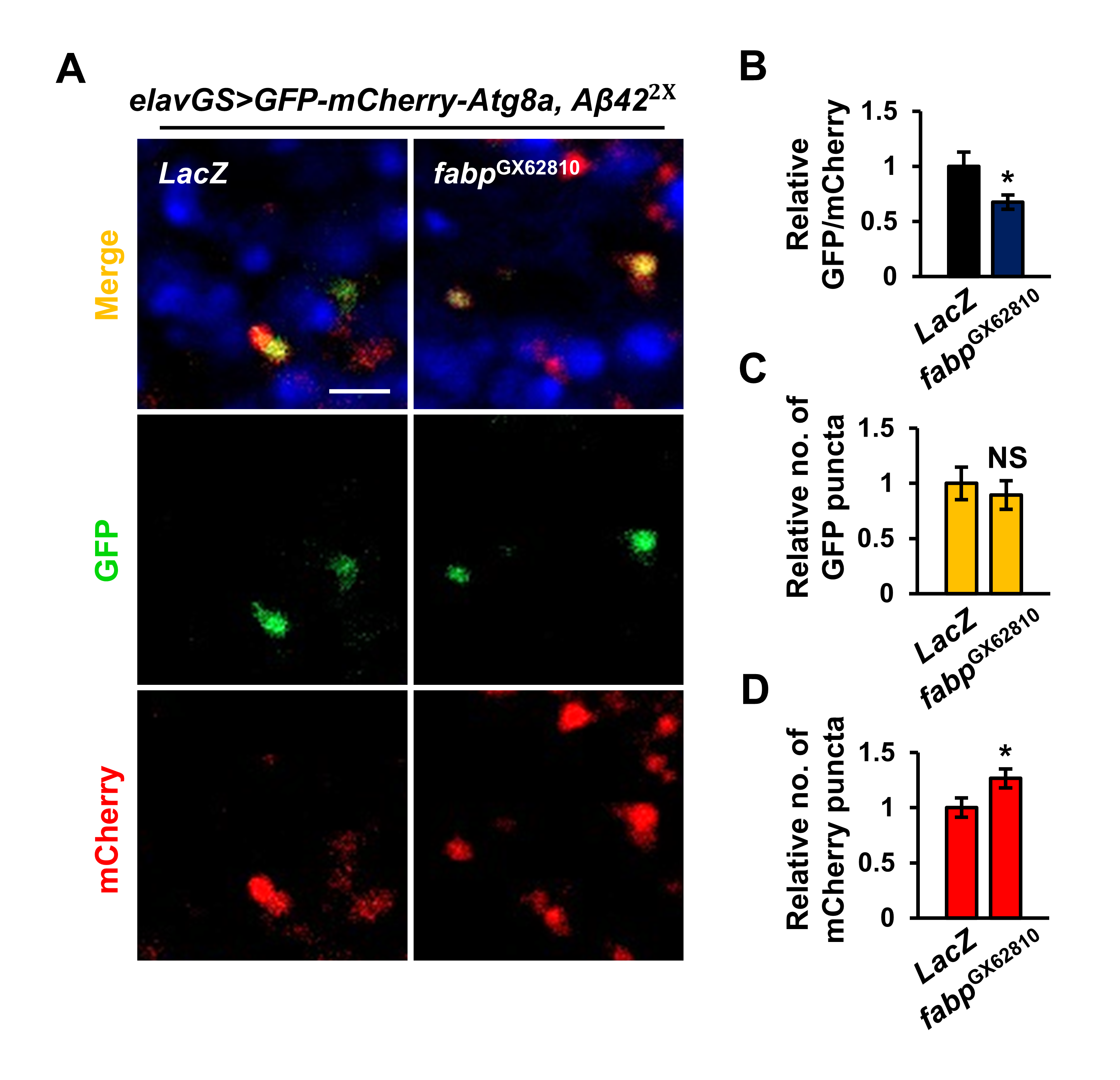

Supplement: S7 Fig — (A) Confocal images showing GFP-mCherry-Atg8a puncta in the brains of control flies (elavGS>GFP-mCherry-Atg8a, Aβ422x/LacZ) and Aβ42-expressing flies with fabp overexpression (elavGS>GFP-mCherry-Atg8a, Aβ422x/fabpGX62810). (B-D) Quantification of the relative ratio of GFP to mCherry puncta (B) and the number of GFP (C) and mCherry (D) puncta (Student’s t-test, n ≥ 8, *p < 0.05, NS, not significant). All data are expressed as mean ± SEM. Scale bar: 2 μm. (TIF) [file pgen.1011475.s024.TIF]

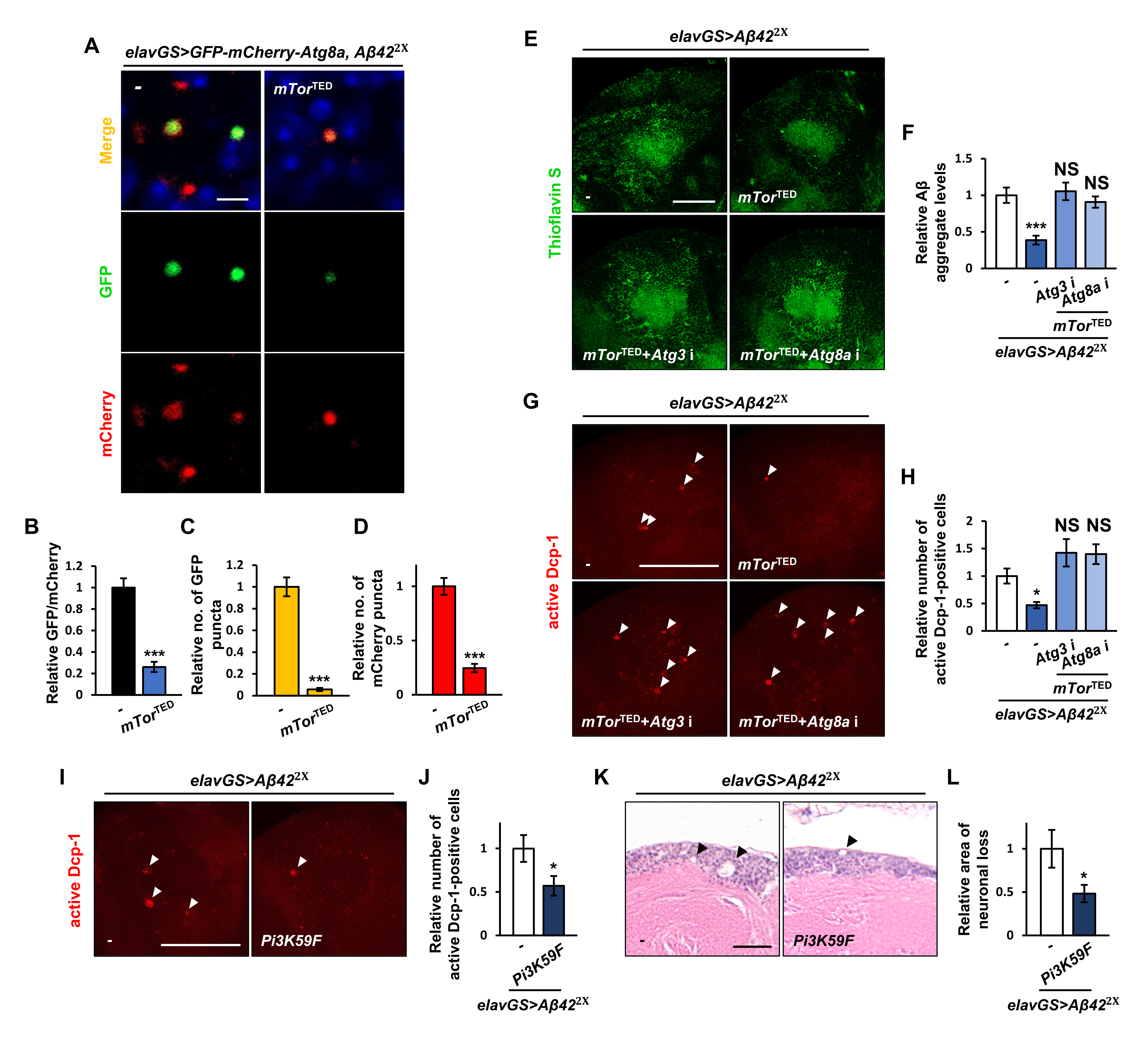

Supplement: S8 Fig — (A) Confocal images showing the GFP-mCherry-Atg8a puncta in the brains of flies with overexpression of mTor dominant negative form (mTorTED). Blue dots indicate DAPI-stained nuclei. (B-D) Quantification of the relative ratio of GFP to mCherry puncta (B), the number of GFP (C), and mCherry (D) puncta in the brains of indicated flies (Student’s t-test, n ≥ 12, ***p < 0.001). (E-H) Effect of mTorTED expression with or without Atg3 i or Atg8a i expression on Aβ aggregation (E, F) and Aβ-induced apoptosis (G, H). (E, G) Confocal images of the brains showing the thioflavin S (E) or active Dcp-1 (G) staining. (F, H) Quantification of the Aβ aggregate intensity (F) or the relative number of active Dcp-1-positive cells (H) in the brains of indicated flies (one-way ANOVA test, *p < 0.05, ***p < 0.001, NS, not significant; F, n ≥ 10; H, n ≥ 6). (I-L) Effects of Pi3K59F expression on Aβ-induced apoptosis (I, J) and neurodegeneration (K, L). (I) Confocal images showing apoptotic cells in the brains of indicated flies. (J) Quantification of the relative number of active Dcp-1-positive cells of indicated flies (Student’s t-test, n = 10, *p < 0.05). (K) Representative images showing the H&E-stained frontal brain sections of indicated flies. (L) Quantification of the relative area of vacuoles in the brains of indicated flies (Student’s t-test, n ≥ 16, *p < 0.05). All data are expressed as mean ± SEM. Scale bars: 2 μm (A), 20 μm (K), 50 μm (E), and 100 μm (G, I). White arrow heads indicate active Dcp-1-positive cells and black arrow heads indicate vacuoles. (TIF) [file pgen.1011475.s025.TIF]
